# Supplementary material for: Correlative 3D microscopy of single cells using super-resolution and scanning ion-conductance microscopy
Source: Nat Commun. 2021 Jul 27;12:4565. doi: 10.1038/s41467-021-24901-3 (PMC8316521; doi:10.1038/s41467-021-24901-3)
Supplement: Supplementary file 1 — Supplementary Information [file 41467_2021_24901_MOESM1_ESM.pdf]

Correlative 3D microscopy of single cells using super-resolution and scanning ion-conductance microscopy: Supplementary information

Vytautas Navikas[1,7], Samuel M. Leitao[2,7], Kristin S. Grussmayer[1,6], Adrien Descloux[1], Barney Drake[2], Klaus Yserentant[3], Philipp Werther[4], Dirk-Peter Herten[3], Richard Wombacher[4,5], Aleksandra Radenovic[1]*, Georg E. Fantner[2]*

[1]Laboratory of Nanoscale Biology, Institute of Bioengineering, School of Engineering, Swiss Federal Institute of Technology Lausanne (EPFL), Lausanne, Switzerland. [2]Laboratory for Bio- and Nano-Instrumentation, Institute of Bioengineering, School of Engineering, Swiss Federal Institute of Technology Lausanne (EPFL), Lausanne, Switzerland. [3]College of Medical and Dental Sciences, Medical School & School of Chemistry, University of Birmingham, Birmingham, United Kingdom. [4]Institute of Pharmacy and Molecular Biotechnology, Heidelberg University, Heidelberg, Germany. [5]Department of Chemical Biology, Max Planck Institute for Medical Research, Heidelberg, Germany. [6]Current address: Grussmayer Lab, Department of Bionanoscience, Faculty of Applied Science and Kavli Institute for Nanoscience Delft, Delft University of Technology, Delft, Netherlands. [7]These authors contributed to manuscript equally: Vytautas Navikas, Samuel M. Leitao. *e-mail: georg.fantner@epfl.ch and aleksandra.radenovic@epfl.ch

Supplementary information content

Supplementary figures 1- 21 and a Supplementary Table.

List of supplementary figures:

1. SICM image of a supported lipid bilayer for axial resolution estimation.
2. SOFI resolution estimation for two-color 2D and 3D SOFI images.
3. Labelling density and bleaching kinetics of self-blinking dyes.
4. High order SOFI comparison with SMLM.
5. Multiple two-color 4th order SOFI images.
6. SICM SOFI image co-registration procedure.
7. Comparison of image quality of 3D SOFI orders.
8. Multiple correlative SICM and two-color 3rd order 3D SOFI images.
9. SICM topographical map of microtubules.
10. SICM setup with an environmental control chamber for live cell imaging.
11. Live-cell SICM-SOFI imaging of actin of filopodia for 42 min.
12. Live-cell SICM-SOFI imaging of a single cell.
13. Cross sections used to calculate Pearson-correlation coefficient between different channels.
14. Detailed schematics of 2D SOFI setup.
15. Detailed schematics of 3D SOFI setup.
16. SEM characterization of glass nanocapillaries.
17. Detailed schematics of a combined SICM-SOFI setup.
18. Coverslip fabrication and binary mark map generation.
19. Phalloidin-f-HM-SiR chemical synthesis.
20. Kinetics of self-blinking dyes for high-order SOFI imaging and ON time estimation.
21. Description of final 3D SOFI data visualization in Blender 3D.

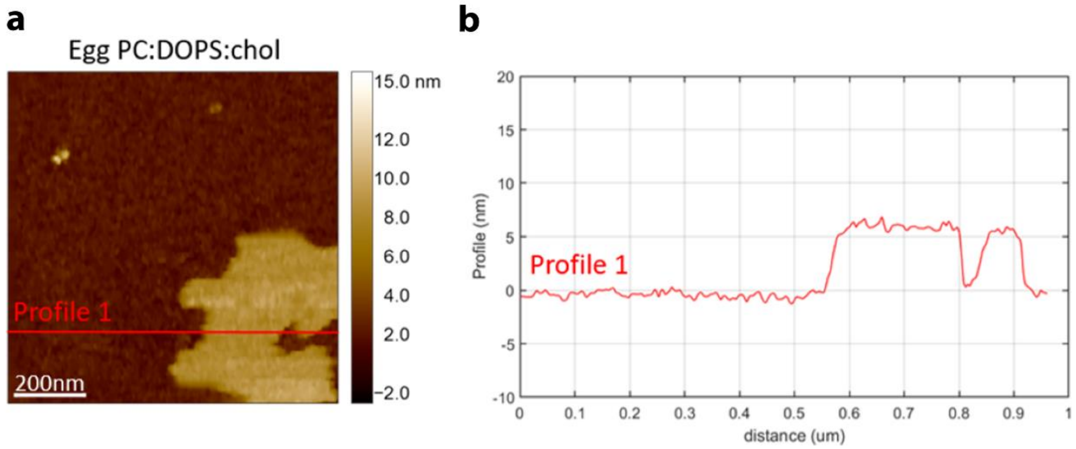

Supplementary Figure 1. SICM image of a supported lipid bilayer (SLB) for axial resolution estimation. (a) SICM image of a SLB, composed of Egg PC:DOPC:chol (3:1:2), deposited on mica acquired using 15 nm radius nanocapillary. Red line shows a location of height profile displayed in (b). 480 x 480 pixels image acquired at pixel acquisition rate of 300 Hz with a hopping height of 100 nm. (b) Height profile of the SLB indicating the axial resolution of SICM below 5 nm. 15nm radius pipette was used for imaging. This figure is representative of more than 3 SICM images from at least 2 independent experiments.

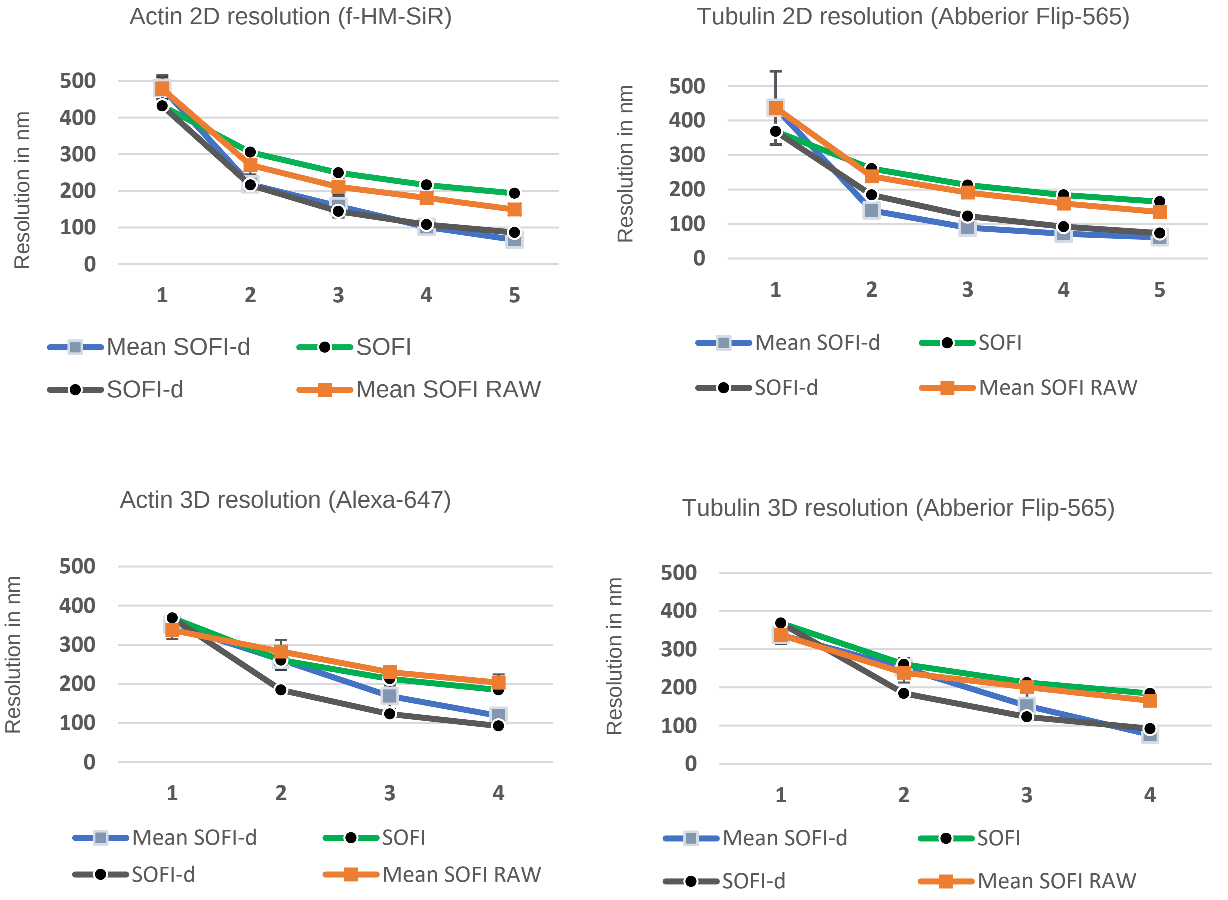

Supplementary Figure 2. SOFI resolution estimation for two-color 2D and 3D SOFI images. Resolution was estimated with a parameter-free image resolution estimation algorithm [1]. For 2D images, each value is calculated from 8 2D SOFI images from 8 separate cells. For 3D SOFI stack resolution was estimated in all planes (N=8, N=15, N=22, N=29) for corresponding SOFI orders from the image volume displayed in Figure 3 and are plotted as mean ± s.d. Theoretical values for SOFI and SOFI-d (deconvolved) based on the diffraction limited point-spread function size are also plotted.

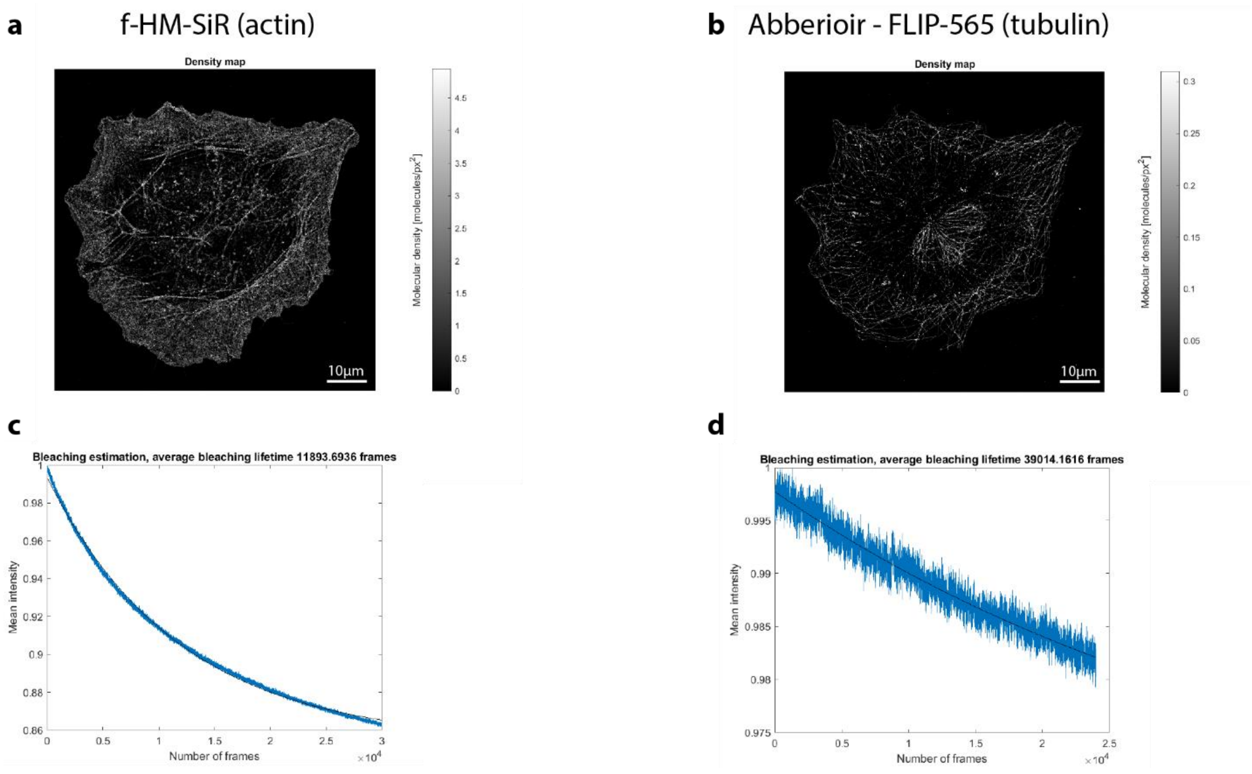

Supplementary Figure 3. Labelling density and bleaching kinetics of self-blinking dyes. Molecular densities of f-HM-SiR (a) and Abberior-FLIP 565 (b) dyes from a representative image, together with bleaching curves (c-d) estimated from the corresponding image stacks. Calculated average bleaching lifetimes (8 stacks with 30 000 frames) for f-HM-SiR dye was 406 ± 168 s and 625 ± 130 s for Abberior FLIP-565 (mean ± s.d, N=8 image stacks consisting of 30 000 frames). This figure is representative of 8 image stacks from at least two independent experiments.

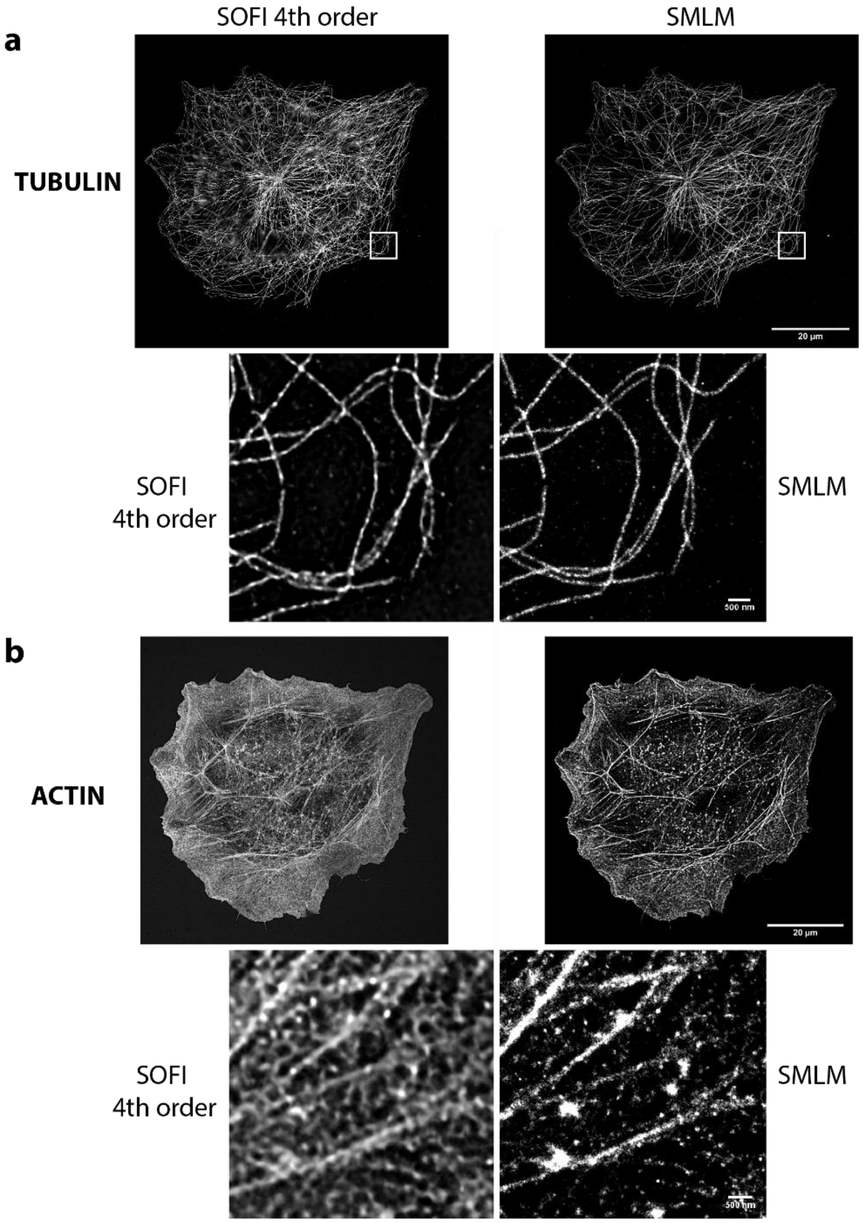

Supplementary Figure 4. High order SOFI comparison with SMLM. a) 4th order SOFI and SMLM (Thunderstorm) image quality comparison. Images stacks were processed with the latest version of Thunderstorm software[2] by using single-emitter fitting function. It is visible that in sparse blinking conditions (Abberior FLIP-565 labelled microtubules) both approaches (SOFI and Thunderstorm) perform similarly, however for f-HM-SiR labelled f-actin SMLM seems to produce localization artifacts, that are expected for high-density data. Resolutions metric stated in the paper were computed with image decorrelation analysis algorithm[1]. The localization precision might be improved by using multi-emitter fitting or pre-processing tools such as HAWK[3], however this is clearly outside of the scope for this study. This figure is representative of more than 3 SOFI/SMLM single-cell images.

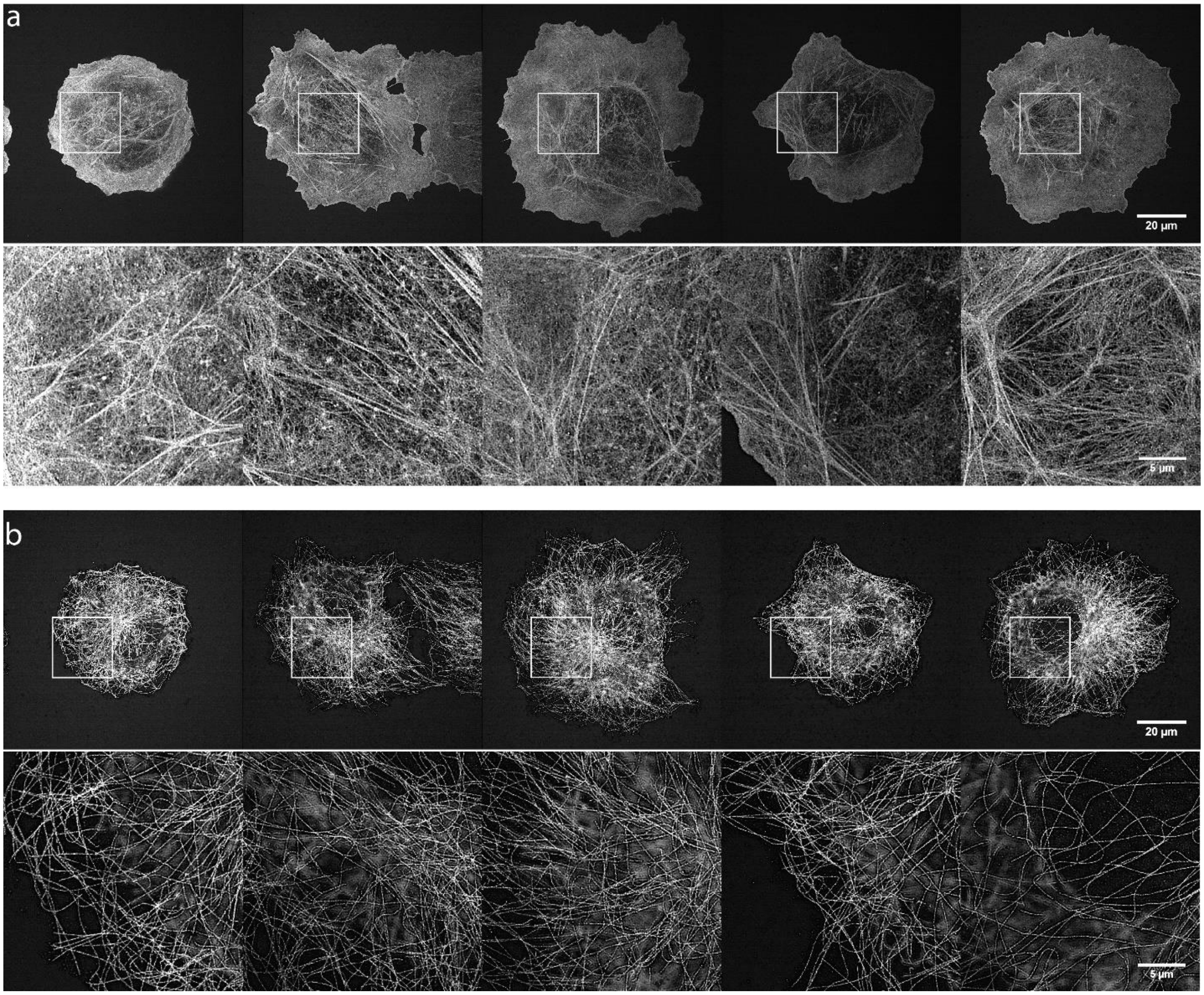

Supplementary Figure 5. Multiple two-color 4th order SOFI images. 4th order SOFI images of phalloidin-f-HM-SiR labeled actin (a) and Abberior FLIP-565 immunostained tubulin (b). Zoom-ins of 25 µm are shown together with the whole 90x90 µm field of view images. This figure is representative of more than 10 correlative 2D SOFI single-cell images obtained from at least two independent experiments.

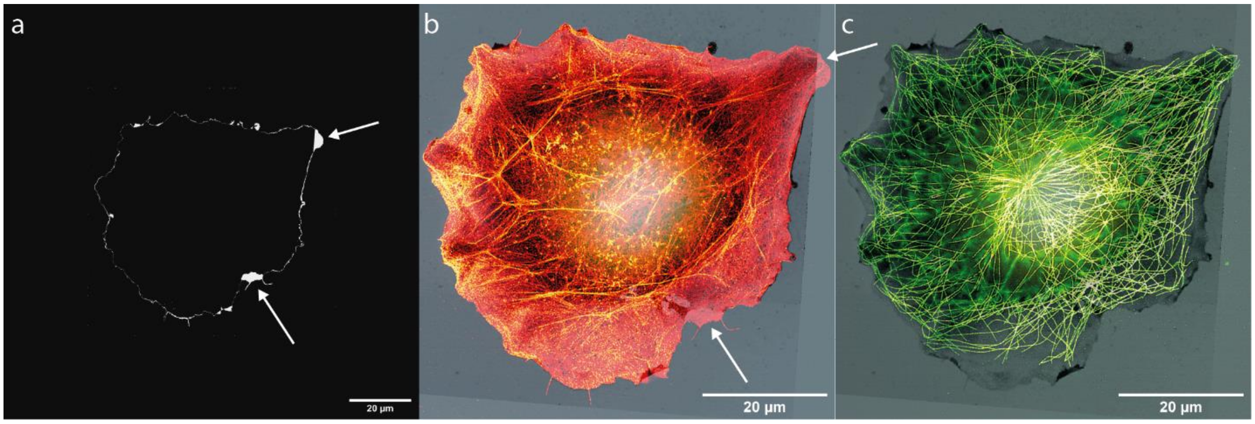

Supplementary Figure 6. SICM SOFI image co-registration procedure. (a) Difference of the tresholded SICM and actin channels revealing the different cell features between SICM and actin channels. (b) SICM image overlaid with actin and (c) channels. Arrows are showing the ambiguities marked in image (a). 2D SOFI images were aligned with SICM topographical map based on the actin channel. Same features were manually depicted in both images and affine transformation matrix was computed using at least 10 corresponding marks. 2D tubulin image was transformed using the same transformation matrix assuming that the lateral drift between two SOFI images is neglectable. 3D SOFI image stacks were processed in the same way expect in this case two-color stacks were aligned based on the brightfield microscopy by phase correlation using the images recorded before acquiring each of the stack. This figure is representative of more than 10 correlative 2D SICM/SOFI single-cell images obtained from at least two independent experiments.

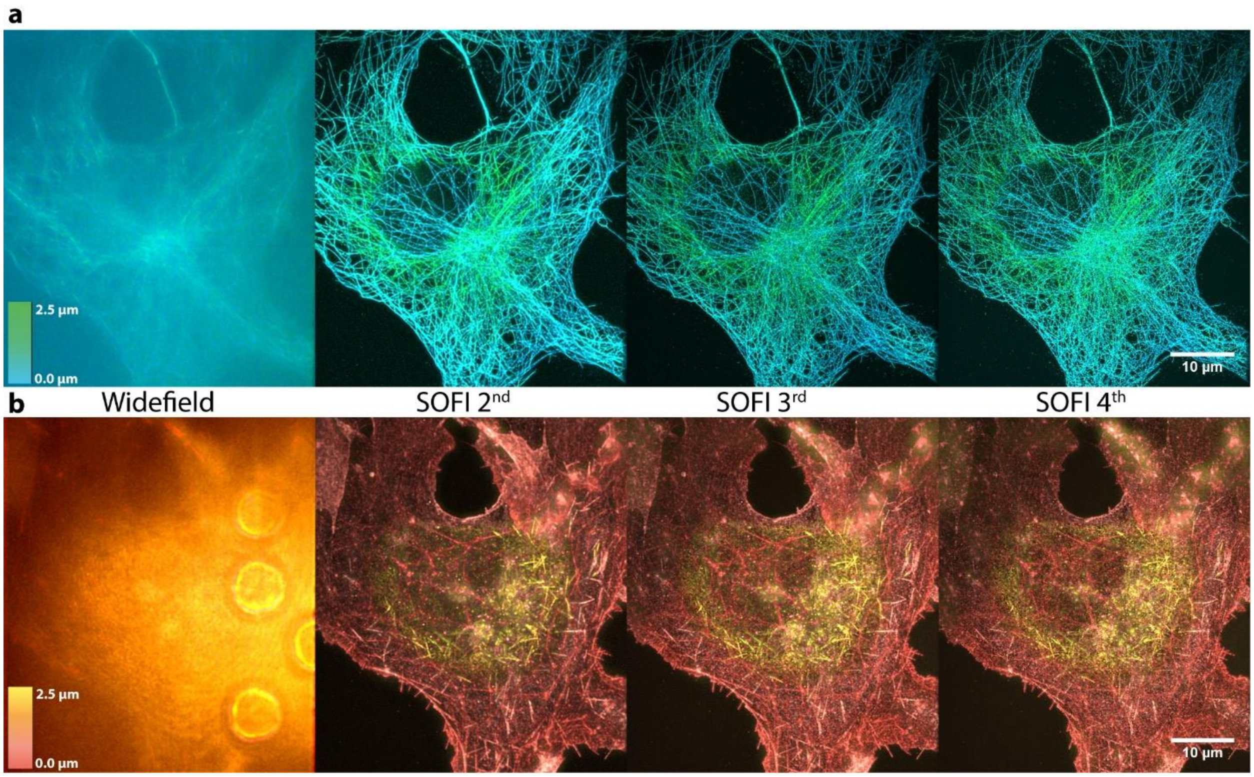

Supplementary Figure 7. Comparison of image quality of 3D SOFI orders. SOFI orders for the two-color 3D SOFI image used in the main text Figure 3. The height scales are indicated. This figure is representative of more than 10 two-color 3D SOFI images obtained from at least two independent experiments.

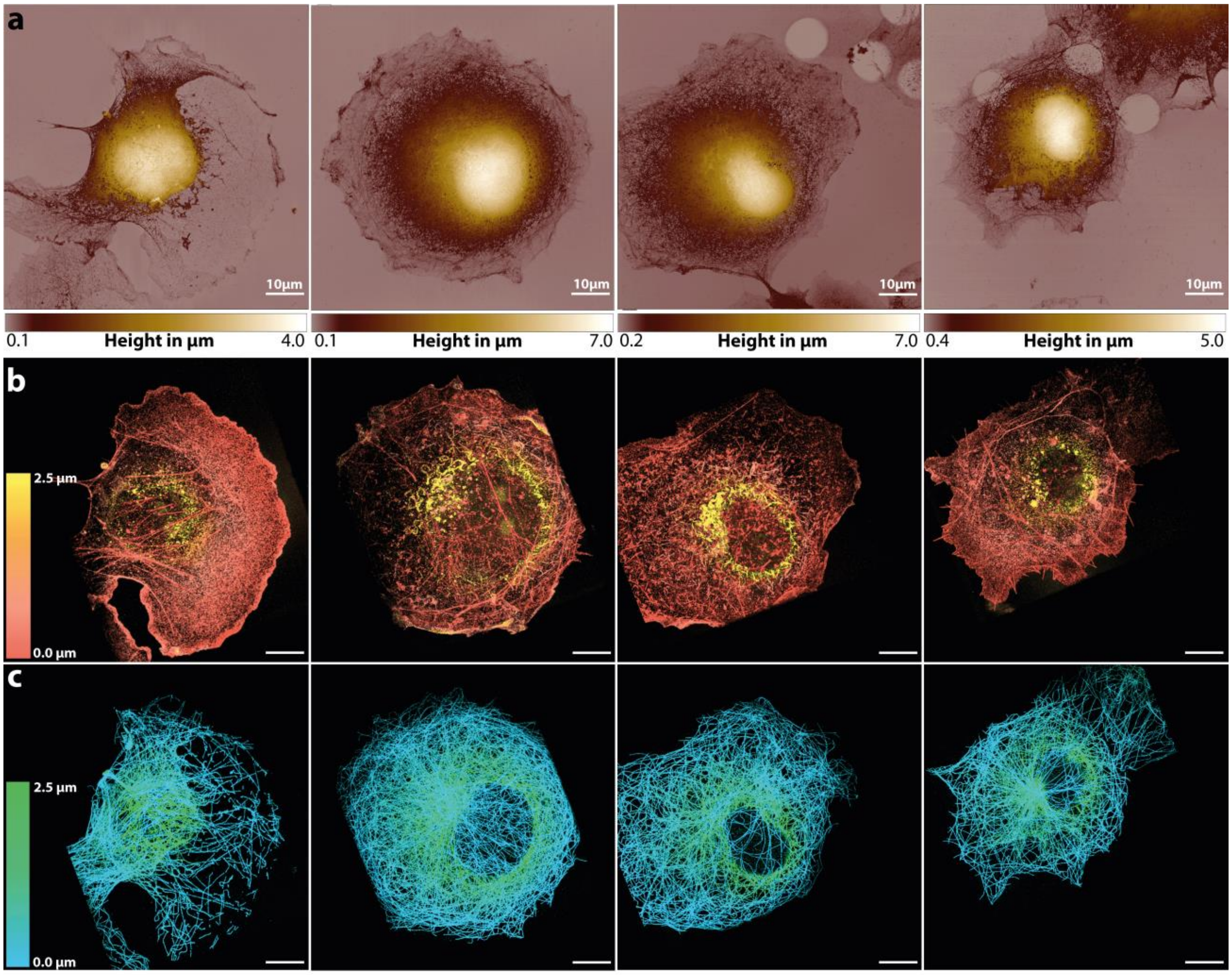

Supplementary Figure 8. Multiple correlative SICM and two-color 3rd order 3D SOFI images. (a) SICM images (1024 x 1024 pixels, 78nm pixel size), acquired at pixel acquisition rate of 200 Hz with a hopping height of 5 µm; together with a coaligned 3rd order SOFI images of phalloidin-Alexa647 labeled actin (b)) and Abberior FLIP-565 labelled tubulin (c). Axial scales are represented as color bars. Scale bars are 10 µm in all images. All images were correlated according to a procedure decribed in Methods section and in (Supplementary Figure 6). This figure is representative of more than 10 correlative SICM and 2-color 3D-SOFI single-cell images obtained from at least two independent experiments.

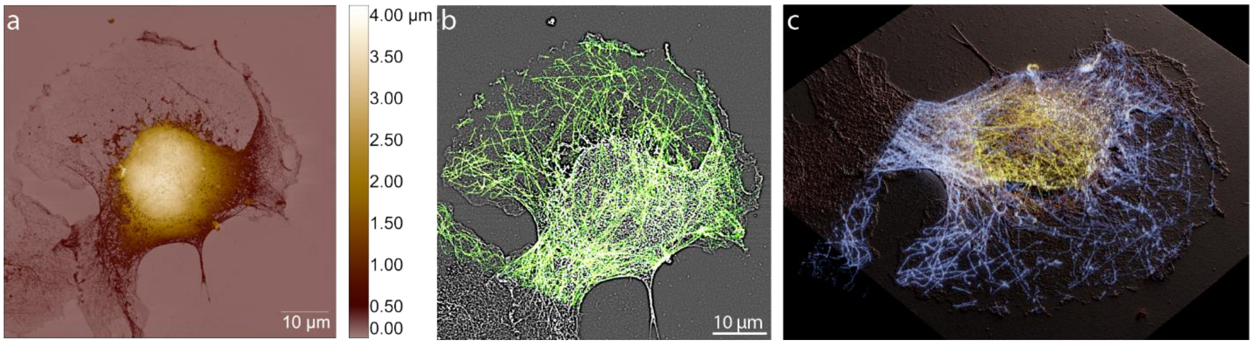

Supplementary Figure 9. SICM topographical map of microtubules. (a) SICM image of a single cell which has part of the membrane removed by permeabilization with Triton X-100. Interestingly, this allows to reveal a preserved microtubular network of the inner part of the cell, which can be better resolved by further spatially filtering the SICM image (b) which is overlaid with a tubulin fluorescence signal from the bottom plane of 3rd order 3D SOFI stack. Bandpass spatial filter with 2-8 px range was used applied on the image (a). (c) Final 3D rendering in Blender 3D software with an overlaid SOFI and SICM data. This figure is representative of more than 3 correlative SICM/SOFI single-cell images.

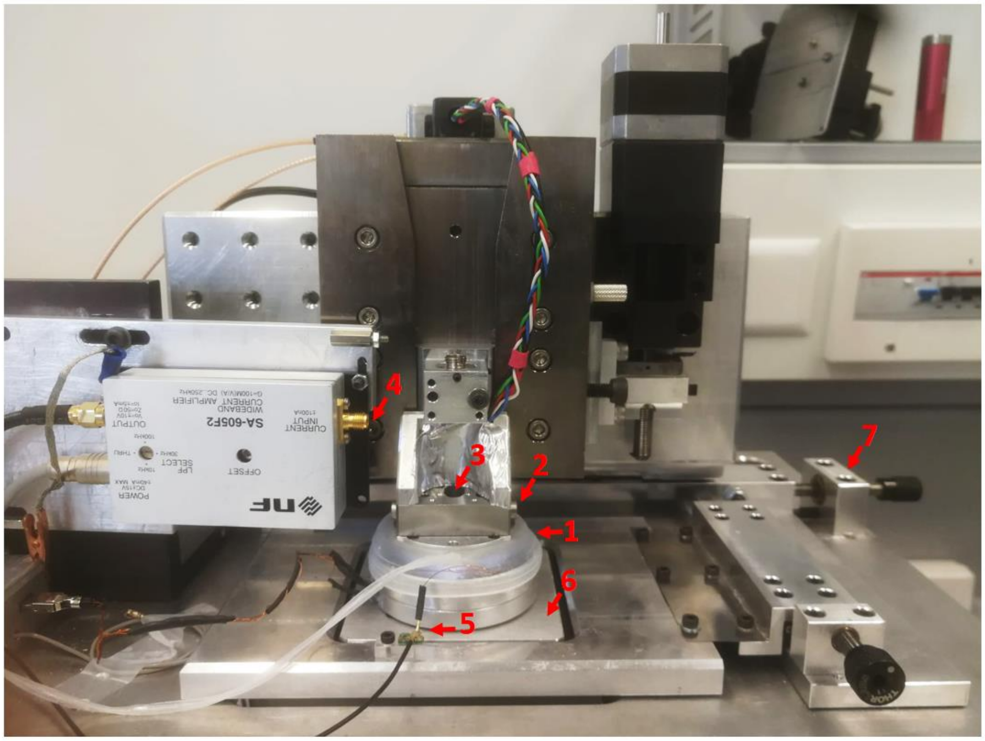

Supplementary Figure 10. SICM setup with an environmental control chamber for live cell imaging described in detail previously[4]. 1) Mini-incubator for environmental control. 2) Pipette actuator. 3) Pipette inlet. 4) Transimpedance amplifier input for the pipette electrode. 5) Bath electrode. 6) XY scanner. 7) Translation stage.

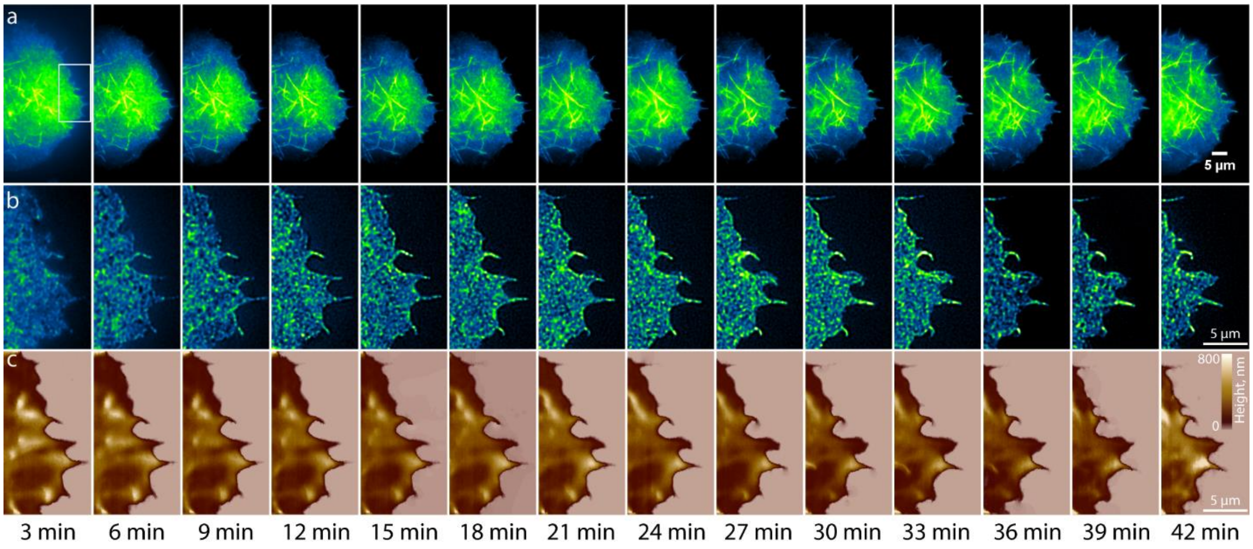

Supplementary Figure 11. Live-cell SICM-SOFI imaging of actin of filopodia for 42 min. Acquisition was performed consequently by recording 10x20 µm SICM scans and 300 frame long fluorescence stacks for imaging photoactivated Lifeact-mEOS-2. Standard deviation image sequence (a) and 2nd order SOFI (b) aligned with SICM topography images (c) are shown. COS-7 cells were transfected as described in the Methods section by using a Lifeact-mEOS-2 plasmid and imaged after 24 h in FluoroBrite medium. 200 W/cm^2 of 561 imaging laser and 0.2 W/cm^2 of 405 nm activation laser were used for illumination with a corresponding exposure time of 50 ms. Corresponding SICM height maps were recorded at 200 x 100 pixels image (100nm pixel size), acquired at a pixel acquisition rate of 200 Hz with a hopping height of 1 µm. This time-lapse sequence is representative of more than 5 independent correlative SICM/SOFI time-lapse experiments.

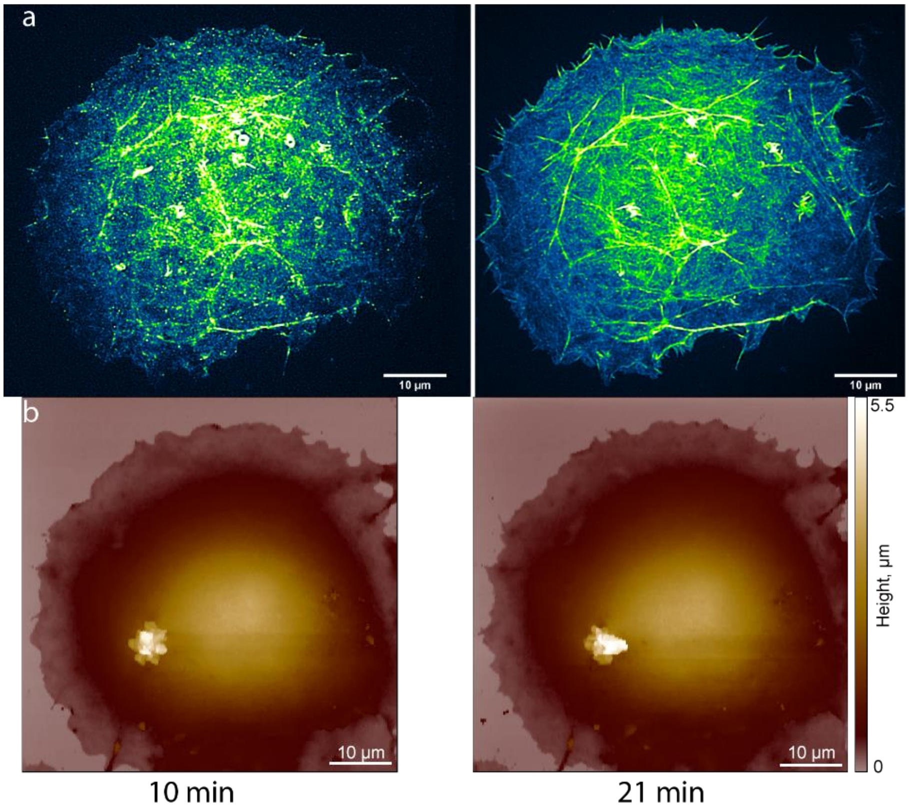

Supplementary Figure 12. Live-cell SICM-SOFI imaging of a single cell. Acquisition was performed consequently by recording 60 x 60 µm SICM scans and 2 fluorescence stacks (300 and 1000 frames long) of photoactivated mEOS-2 for 2nd order SOFI computation. 2nd order SOFI (a) aligned with SICM topography images (b) are showed. COS-7 cells were transfected as described in the Methods section by using a Lifeact-mEOS-2 plasmid and imaged after 24 h in FluoroBrite medium. 200 W/cm^2 of 561 imaging laser and 0.2 W/cm^2 of 405 nm activation laser were used for illumination with a corresponding exposure time of 50 ms. Corresponding SICM height maps recorded at 512 x 256 pixels image (117nm pixel size), acquired at a pixel acquisition rate of 200 Hz with a hopping height of 3 µm. White spot in the SICM image is most likely a particle, attached from the solution. This time-lapse sequence is representative of more than 3 independent correlative SICM/SOFI time-lapse experiments while imaging the whole cell.

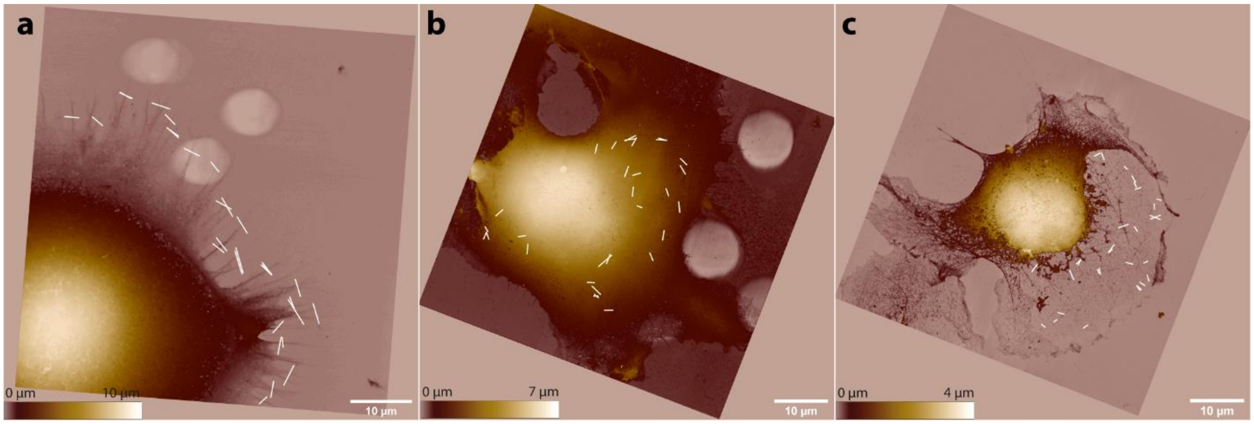

Supplementary Figure 13. Cross sections used to calculate Pearson-correlation coefficient between different channels. For Fig 5f 1D cross sections were manually depicted by selecting the structures of interest by hand. Filopodia (a), microvilli (b) and microtubules (c) were selected. Height scales are indicated. This figure is representative of more than 10 correlative SICM and 2-color 2D/3D SOFI single-cell images obtained from at least two independent experiments.

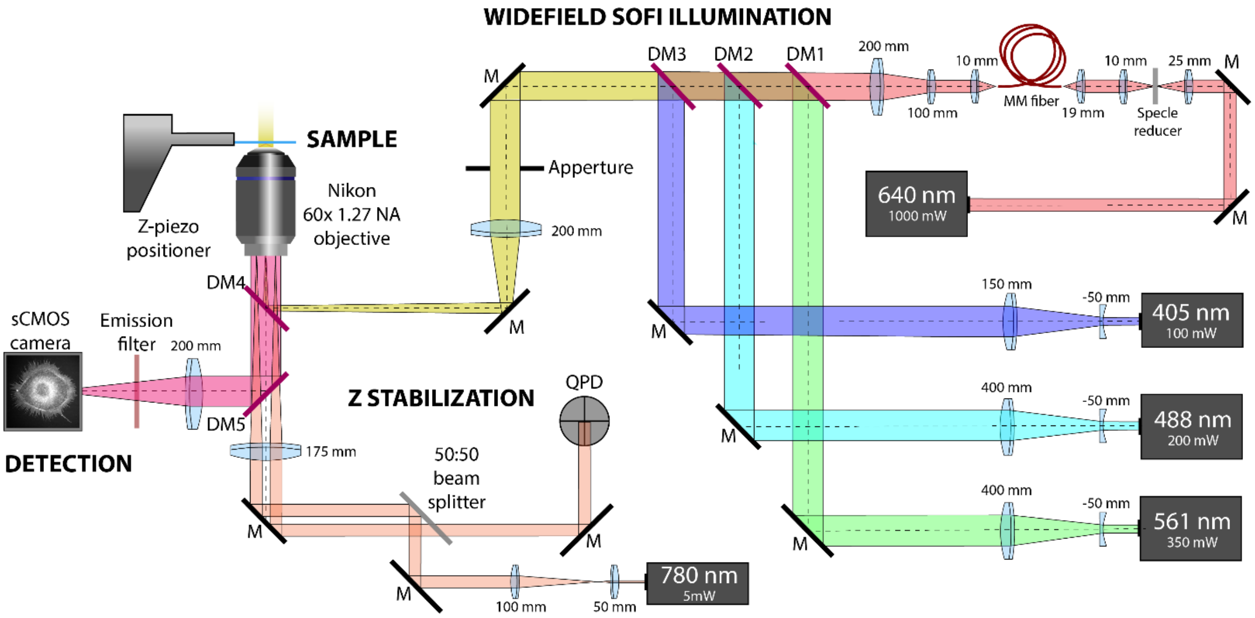

Supplementary Figure 14. Detailed schematics of 2D SOFI setup. Schematics of the setup used for 2D SOFI imaging as described in the Methods section. M – mirror, DM – dichroic mirror, MM – multi-mode, QPD – quadrant phodiode.

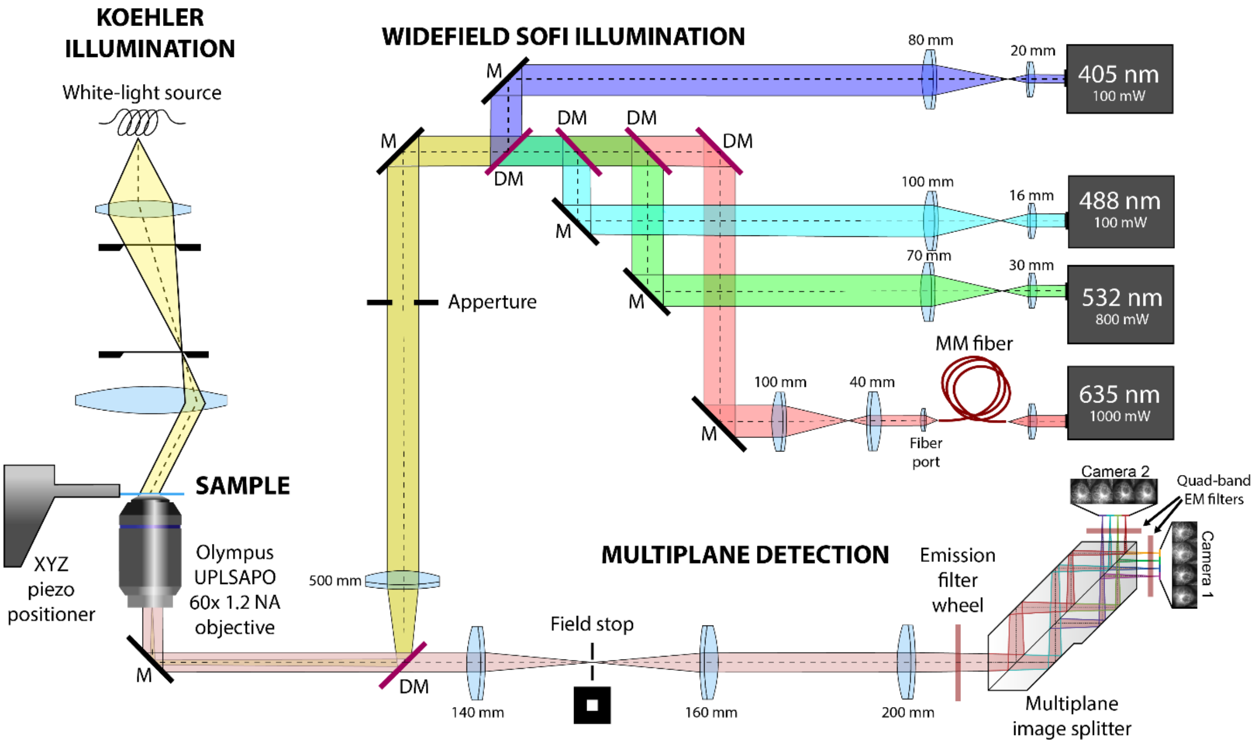

Supplementary Figure 15. Detailed schematics of 3D SOFI setup. Schematics of the setup used for 3D SOFI imaging as described in the Methods section. M – mirror, DM – dichroic mirror, MM – multi-mode, QPD – quadrant phodiode, EM – emission.

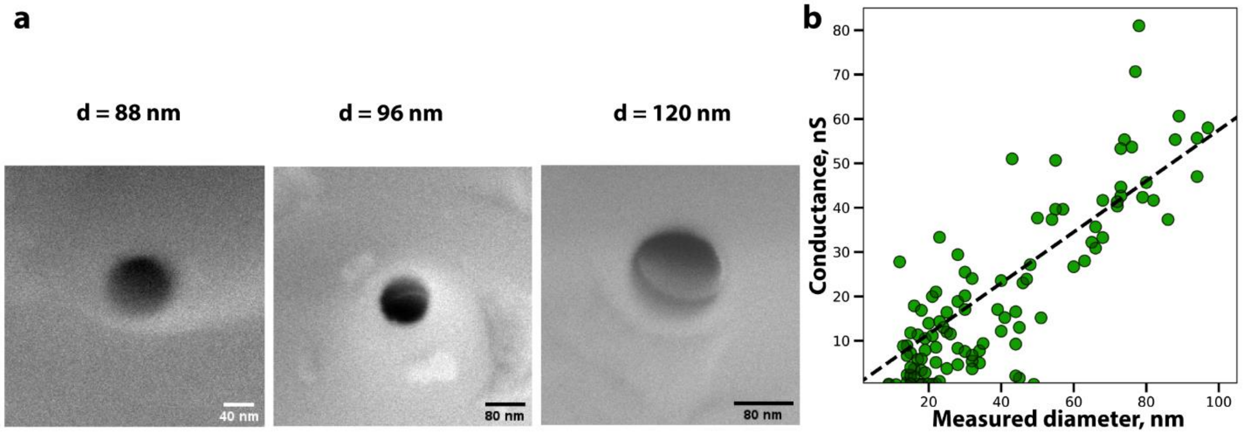

Supplementary Figure 16. Characterization of glass nanocapillaries. (a) Selected scanning electron microscope (SEM) images of glass nanocapilaries without conductive coating. These SEM images are representative of more than 100 SEM images used to calculate the diameters of the pipettes plotted in

(b)(b) Conductance measured in 400 mM KCl solution vs opening diameter measured with SEM (N=138). The dashed line represents a least-square fit of the following equation: $G = \sigma\left(\frac{4t}{\pi D d} + \frac{1}{2D} + \frac{1}{2d}\right)^{-1}$, where *D* is a shaft diameter (400 µm), *d* is the diameter of the nanopore and *t* is the taper length, which was determined to be 2 mm. This model was established in a previous study[5]. The figure is composed from the data published in our previous study[6].

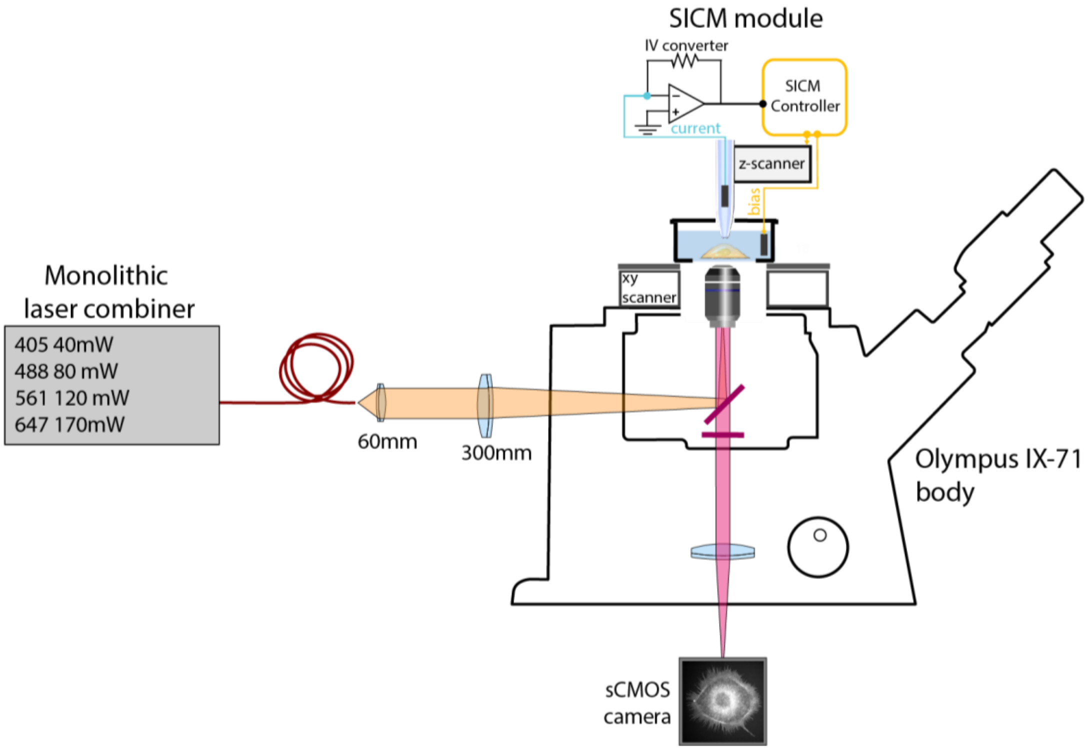

Supplementary Figure 17. Detailed schematics of a combined SICM-SOFI setup.

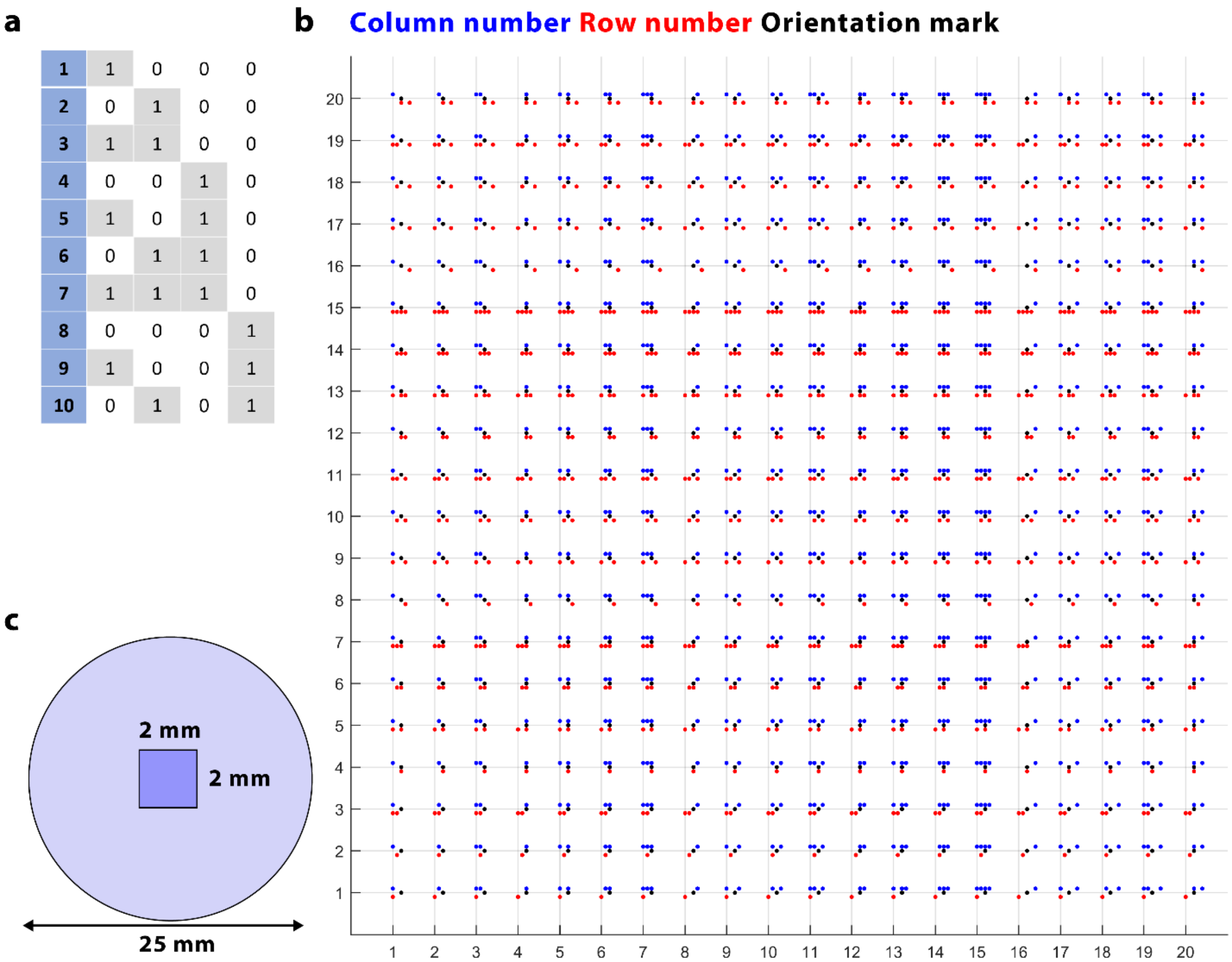

Supplementary Figure 18. Coverslip fabrication and binary mark map generation. (a) The principle of number representation in binary values. 4 binary digits were used to represent numbers from 1 to 20. (b) The final layout of the sample map. Layout was generated with a Matlab code, by using the dec2bin function. Binary numbers were used for x and y axis. The static dot in the middle was incorporated for a better determination of the sample orientation. (c) Schematics of the dimensions of the binary digit pattern.

Supplementary Figure 19. Phalloidin-f-HM-SiR chemical synthesis. Reaction scheme of Phalloidin-f-HM-SiR conjugate.

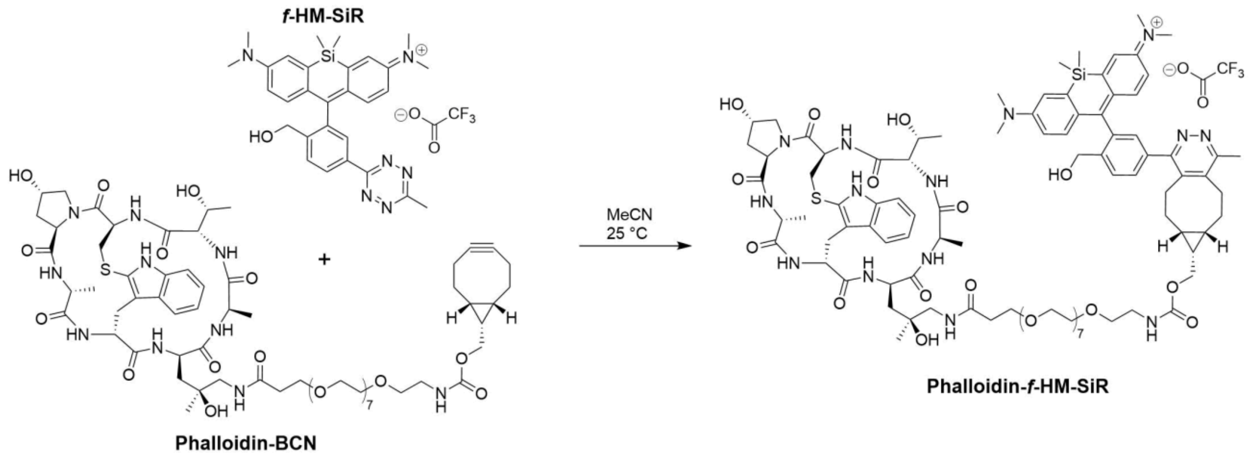

Phalloidin-*f*-HM-SiR was synthesized from *f*-HM-SiR[7] and Phalloidin-BCN [8] [9], which were prepared according to literature procedures. In short: Phalloidin-BCN (46 µg) was dissolved in anhydrous Acetonitrile (MeCN) (90 µL) and *f*-HM-SiR (24.8 µg, 4 µL from 10 mM stock solution) was added at room temperature. The reaction mixture was incubated for 4 h in a thermo-shaker at 700 rpm and subsequently purified by High Performance Liquid Chromatography (HPLC) (gradient 20-90% solvent B / solvent A; in 40 min). Phalloidin-*f*-HM-SiR was afforded as blue solid and after photometric determination of the amount of substance [10], a 1 mM stock solution in anhydrous dimethyl sulfoxide (DMSO) was prepared. High Resolution Mass Spectrometry (HRMS) (ESI^+) m/z 1867.9047 calculated for $[C_{94}H_{131}N_{14}O_{22}SSi]^+$ (M^+), 1867.9073 found; m/z 945.4470 calculated for $[C_{94}H_{131}N_{14}NaO_{22}SSi]^{2+}$ (M^++Na^+), 945.4482 found. HPLC analytics and semi-preparative purifications were conducted on an Agilent 1100 series HPLC system. Phenomenex Luna 3 µ and 5 µ C18 reversed-phase columns were used for these purposes (Solvent A: H_2O containing 0.1% trifluoroacetic acid (TFA); Solvent B: MeCN containing 0.1% TFA). Collected HPLC fractions were dried by lyophilization. Mass spectrometry was performed on a Bruker microTOF-QII electrospray ionization mass spectrometer (ESI-MS).

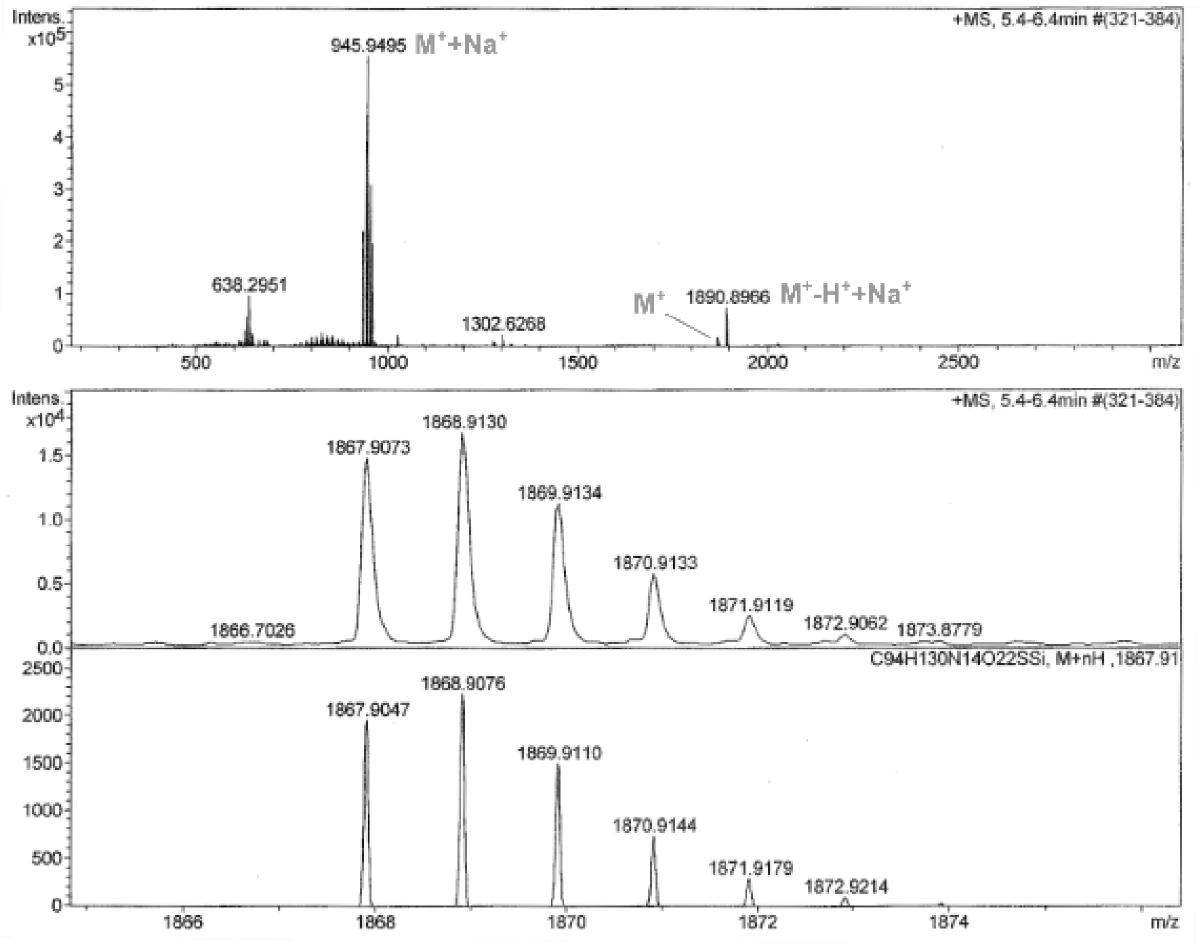

HRMS (ESI$^+$) of Phalloidin-*f*-HM-SiR

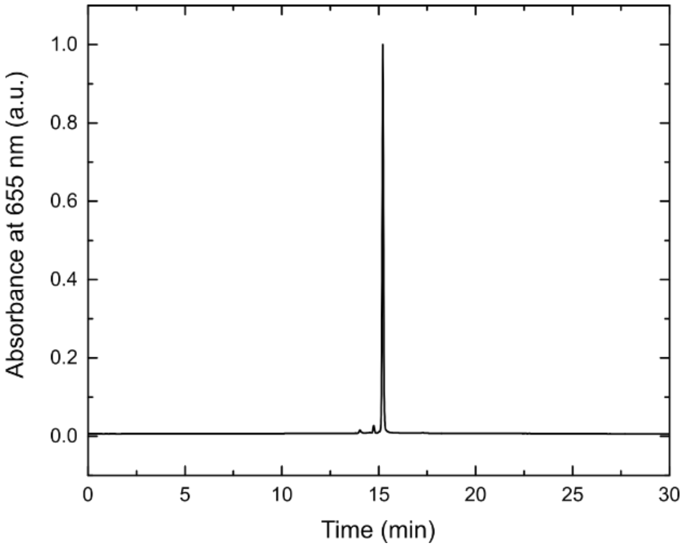

HPLC chromatogram of Phalloidin-*f*-HM-SiR

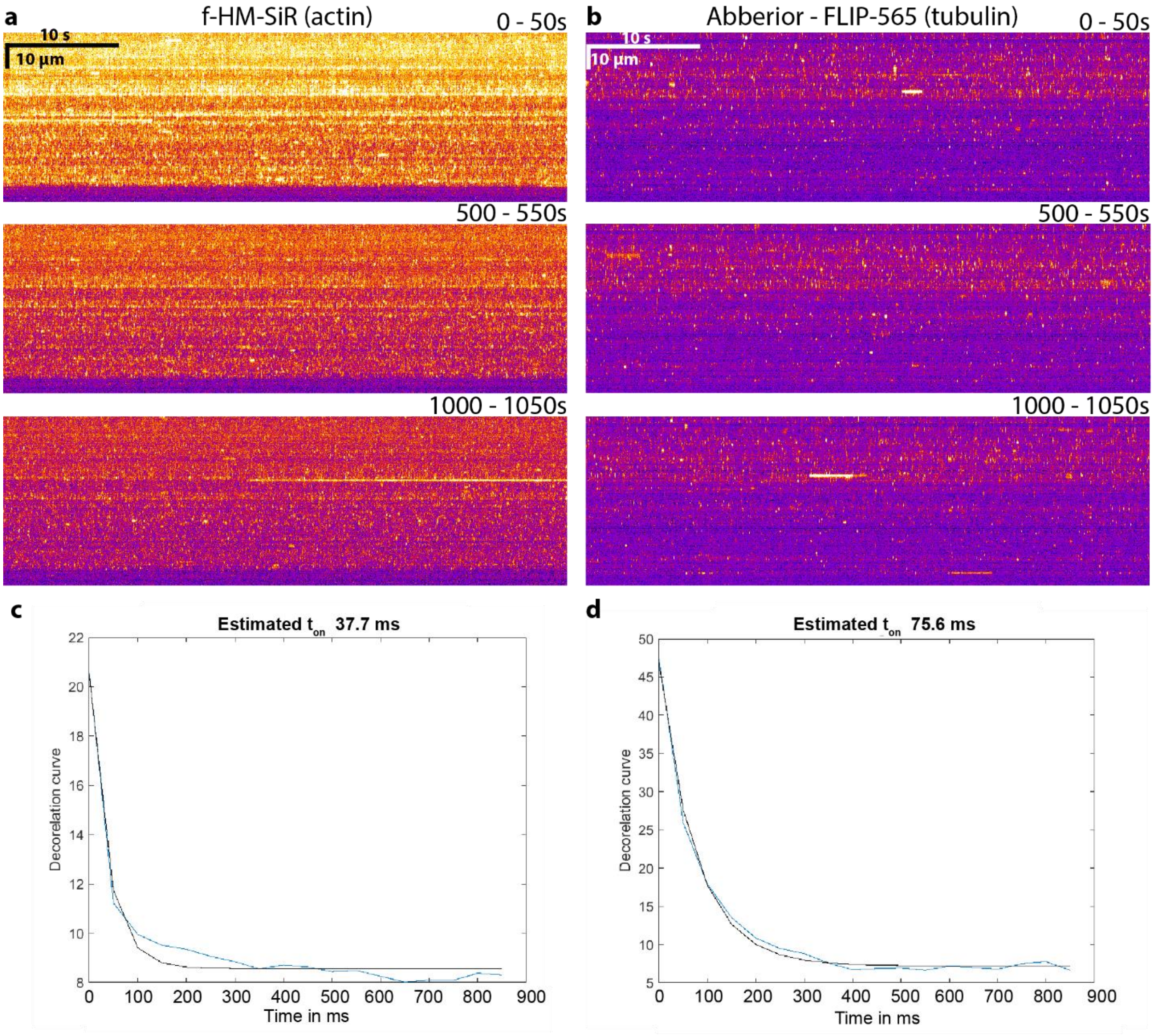

Supplementary Figure 20. Kinetics of self-blinking dyes for high-order SOFI imaging and ON time estimation. Kymographs of signal intensity over time for f-HM-SiR (a) and Abberior-FLIP 565 (b) dyes showing the signal fluctuation over a long-term imaging. Average ON-times were also estimated by computing 2nd order cumulant as a function of time lag and averaging it for subsequences of 500 frames[11]. Corresponding lag functions for stacks showed in (a-b) are showed below (c-d). Mean on time for f-HM-SiR dye was estimated to be 38.7 ms and for Abberior-FLIP 565 – 65.75 ms. However, the exposure time of 50 ms might be the limiting factor for the precision of ON-time estimation. This figure is representative of 8 image stacks from at least two independent experiments.

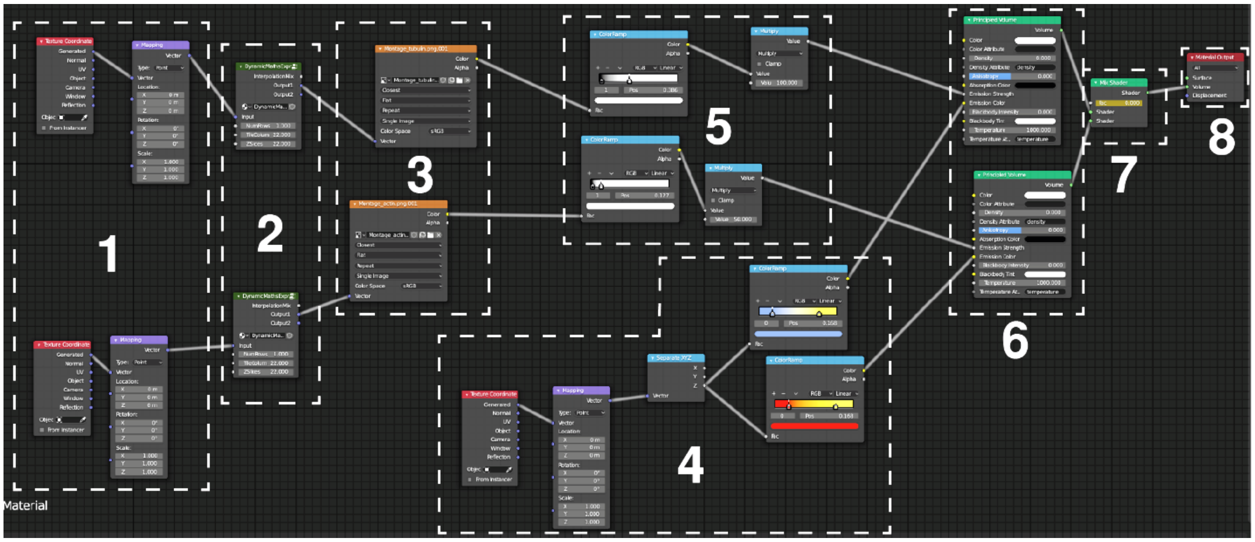

Supplementary Figure 21. Description of final 3D SOFI data visualization in Blender 3D. A simple cube was used as a volume corresponding to the 3D SOFI volume of 2.45x60x60 µm. Two separate colors were visualized in parallel. Different parts of the shader are explained as follows: 1) Setting up the texture coordinates 2) A script node used to make a 3D volume from the stack of SOFI images. Each image is represented as voxel with dimensions determined by a 3D cube 3) Input of a stack file 4) Color ramps used to represent the height in different colors 5) Contrast and intensity adjustment 6) A principled volume shader 7) A mix shader used to switch between tubulin and actin channels 8) A volume output node.

Lifeact-mEOS-2 plasmid sequence (ABP-tdEosFP)
Length: 5410 bp
Reference: Izeddin et al. [12]

ATTAATAGTAATCAATTACGGGGTCATTAGTTCATAGCCCATATATGGAGTTCCGCGTTACATAACTTACGGTAAATGGCCCGCCTGGCTGACCGCCCAACG
ACCCCCGCCCATTGACGTCAATAATGACGTATGTTCCCATAGTAACGCCAATAGGGACTTTCCATTGACGTCAATGGGTGGAGTATTTACGGTAAACTGCCC
ACTTGGCAGTACATCAAGTGTATCATATGCCAAGTACGCCCCCTATTGACGTCAATGACGGTAAATGGCCCGCCTGGCATTATGCCCAGTACATGACCTTAT
GGGACTTTCCTACTTGGCAGTACATCTACGTATTAGTCATCGCTATTACCATGGTGATGCGGTTTTGGCAGTACATCAATGGGCGTGGATAGCGGTTTGACT
CACGGGGATTTCCAAGTCTCCACCCCATTGACGTCAATGGGAGTTTGTTTTGGCACCAAAATCAACGGGACTTTCCAAAATGTCGTAACAACTCCGCCCCATT
GACGCAAATGGGCGGTAGGCGTGTACGGTGGGAGGTCTATATAAGCAGAGCTGGTTTAGTGAACCGTCAGATCCgctagcCACCATGGGCGTGGCCGACCT
GATCAAGAAGTTCGAGAGCATCAGCAAGGAGGAGACTAGTGCCACCATGAGTGCGATTAAGCCAGACATGAAGATCAACCTCCGTATGGAAGGCAACGTA
AACGGGCACCACTTTGTGATCGACGGAGATGGTACAGGCAAGCCTTTTGAGGGAAAACAGAGTATGGATCTTGAAGTCAAAGAGGGCGGACCTCTGCCTT
TTGCCTTTGATATCCTGACCACTGCATTCCATTACGGCAACAGGGTATTCGCCGAATATCCAGACCACATACAAGACTATTTTAAGCAGTCGTTTCCTAAGGG
GTATTCGTGGGAACGAAGCTTGACTTTCGAAGACGGGGGCATTTGCATTGCCAGAAACGACATAACAATGGAAGGGGACACTTTCTATAATAAAGTTCGAT
TTCACGGTGTAAACTTTCCCGCCAATGGTCCAGTTATGCAGAAGAAGACGCTGAAATGGGAGCCCTCCACTGAGAAAATGTATGTGCGTGATGGAGTGCTG
ACTGGTGATATTCGCATGGCTTTGTTGCTTGAAGGAAATGCCCATTACCGATGTGACTTCAGAACTACTTACAAAGCTAAGGAGAAGGGTGTCAAGTTACCA
GGCTACCACTTTGTGGACCACTGCATTGAGATTTTAAGCCATGACAAAGATTACAACAAGGTTAAGCTGTATGAGCATGCTGTTGCTCATTCTGGATTGCCTG
ACAATGCCAGACGATCCGGTAGCGGTCACGGTACTGGTTCTACTGGTTCTGGTTCTTCTGAGAGTGCGATTAAGCCAGACATGAAGATCAACCTCCGTATGG
AAGGCAACGTAAACGGGCACCACTTTGTGATCGACGGAGATGGTACAGGCAAGCCTTTTGAGGGAAAACAGAGTATGGATCTTGAAGTCAAAGAGGGCG
GACCTCTGCCTTTTGCCTTTGATATCCTGACCACTGCATTCCATTACGGCAACAGGGTATTCGCCGAATATCCAGACCACATACAAGACTATTTTAAGCAGTC
GTTTCCTAAGGGGTATTCGTGGGAACGAAGCTTGACTTTCGAAGACGGGGGCATTTGCATTGCCAGAAACGACATAACAATGGAAGGGGACACTTTCTATA
ATAAAGTTCGATTTCACGGTGTAAACTTTCCCGCCAATGGTCCAGTTATGCAGAAGAAGACGCTGAAATGGGAGCCCTCCACTGAGAAAATGTATGTGCGT
GATGGAGTGCTGACTGGTGATATTCGCATGGCTTTGTTGCTTGAAGGAAATGCCCATTACCGATGTGACTTCAGAACTACTTACAAAGCTAAGGAGAAGGG
TGTCAAGTTACCAGGCTACCACTTTGTGGACCACTGCATTGAGATTTTAAGCCATGACAAAGATTACAACAAGGTTAAGCTGTATGAGCATGCTGTTGCTCAT
TCTGGATTGCCTGACAATGCCAGACGACtgtacaAGTAAGCGGCCGCGACTCTAGATCATAATCAGCCATACCACATTTGTAGAGGTTTTACTTGCTTTAAAAA
ACCTCCCACACCTCCCCCTGAACCTGAAACATAAAATGAATGCAATTGTTGTTGTTAACTTGTTTATTGCAGCTTATAATGGTTACAAATAAAGCAATAGCATC
ACAAATTTCACAAATAAAGCATTTTTTTCACTGCATTCTAGTTGTGGTTTGTCCAAACTCATCAATGTATCTTAAGGCGTAAATTGTAAGCGTTAATATTTTGTT
AAAATTCGCGTTAAATTTTTGTTAAATCAGCTCATTTTTTAACCAATAGGCCGAAATCGGCAAAATCCCTTATAAATCAAAAGAATAGACCGAGATAGGGTTG
AGTGTTGTTCCAGTTTGGAACAAGAGTCCACTATTAAAGAACGTGGACTCCAACGTCAAAGGGCGAAAAACCGTCTATCAGGGCGATGGCCCACTACGTGA
ACCATCACCCTAATCAAGTTTTTTGGGGTCGAGGTGCCGTAAAGCACTAAATCGGAACCCTAAAGGGAGCCCCCGATTTAGAGCTTGACGGGGAAAGCCGG
CGAACGTGGCGAGAAAGGAAGGGAAGAAAGCGAAAGGAGCGGGCGCTAGGGCGCTGGCAAGTGTAGCGGTCACGCTGCGCGTAACCACCACACCCGCC
GCGCTTAATGCGCCGCTACAGGGCGCGTCAGGTGGCACTTTTCGGGGAAATGTGCGCGGAACCCCTATTTGTTTATTTTTCTAAATACATTCAAATATGTATC
CGCTCATGAGACAATAACCCTGATAAATGCTTCAATAATATTGAAAAAGGAAGAGTCCTGAGGCGGAAAGAACCAGCTGTGGAATGTGTGTCAGTTAGGGT
GTGGAAAGTCCCCAGGCTCCCCAGCAGGCAGAAGTATGCAAAGCATGCATCTCAATTAGTCAGCAACCAGGTGTGGAAAGTCCCCAGGCTCCCCAGCAGGC
AGAAGTATGCAAAGCATGCATCTCAATTAGTCAGCAACCATAGTCCCGCCCCTAACTCCGCCCATCCCGCCCCTAACTCCGCCCAGTTCCGCCCATTCTCCGC
CCCATGGCTGACTAATTTTTTTTATTTATGCAGAGGCCGAGGCCGCCTCGGCCTCTGAGCTATTCCAGAAGTAGTGAGGAGGCTTTTTTGGAGGCCTAGGCT
TTTGCAAAGATCGATCAAGAGACAGGATGAGGATCGTTTCGCATGATTGAACAAGATGGATTGCACGCAGGTTCTCCGGCCGCTTGGGTGGAGAGGCTATT
CGGCTATGACTGGGCACAACAGACAATCGGCTGCTCTGATGCCGCCGTGTTCCGGCTGTCAGCGCAGGGGCGCCCGGTTCTTTTTGTCAAGACCGACCTGTC
CGGTGCCCTGAATGAACTGCAAGACGAGGCAGCGCGGCTATCGTGGCTGGCCACGACGGGCGTTCCTTGCGCAGCTGTGCTCGACGTTGTCACTGAAGCG
GGAAGGGACTGGCTGCTATTGGGCGAAGTGCCGGGGCAGGATCTCCTGTCATCTCACCTTGCTCCTGCCGAGAAAGTATCCATCATGGCTGATGCAATGCG
GCGGCTGCATACGCTTGATCCGGCTACCTGCCCATTCGACCACCAAGCGAAACATCGCATCGAGCGAGCACGTACTCGGATGGAAGCCGGTCTTGTCGATC
AGGATGATCTGGACGAAGAGCATCAGGGGCTCGCGCCAGCCGAACTGTTCGCCAGGCTCAAGGCGAGCATGCCCGACGGCGAGGATCTCGTCGTGACCCA
TGGCGATGCCTGCTTGCCGAATATCATGGTGGAAAATGGCCGCTTTTCTGGATTCATCGACTGTGGCCGGCTGGGTGTGGCGGACCGCTATCAGGACATAG
CGTTGGCTACCCGTGATATTGCTGAAGAGCTTGGCGGCGAATGGGCTGACCGCTTCCTCGTGCTTTACGGTATCGCCGCTCCCGATTCGCAGCGCATCGCCT
TCTATCGCCTTCTTGACGAGTTCTTCTGAGCGGGACTCTGGGGTTCGAAATGACCGACCAAGCGACGCCCAACCTGCCATCACGAGATTTCGATTCCACCGC
CGCCTTCTATGAAAGGTTGGGCTTCGGAATCGTTTTCCGGGACGCCGGCTGGATGATCCTCCAGCGCGGGGATCTCATGCTGGAGTTCTTCGCCCACCCTAG
GGGGAGGCTAACTGAAACACGGAAGGAGACAATACCGGAAGGAACCCGCGCTATGACGGCAATAAAAAGACAGAATAAAACGCACGGTGTTGGGTCGTT
TGTTCATAAACGCGGGGTTCGGTCCCAGGGCTGGCACTCTGTCGATACCCCACCGAGACCCCATTGGGGCCAATACGCCCGCGTTTCTTCCTTTTCCCCACCC
CACCCCCCAAGTTCGGGTGAAGGCCCAGGGCTCGCAGCCAACGTCGGGGCGGCAGGCCCTGCCATAGCCTCAGGTTACTCATATATACTTTAGATTGATTTA
AAACTTCATTTTTAATTTAAAAGGATCTAGGTGAAGATCCTTTTTGATAATCTCATGACCAAAATCCCTTAACGTGAGTTTTCGTTCCACTGAGCGTCAGACCC
CGTAGAAAAGATCAAAGGATCTTCTTGAGATCCTTTTTTTCTGCGCGTAATCTGCTGCTTGCAAACAAAAAAACCACCGCTACCAGCGGTGGTTTGTTTGCCG
GATCAAGAGCTACCAACTCTTTTTCCGAAGGTAACTGGCTTCAGCAGAGCGCAGATACCAAATACTGTCCTTCTAGTGTAGCCGTAGTTAGGCCACCACTTCA
AGAACTCTGTAGCACCGCCTACATACCTCGCTCTGCTAATCCTGTTACCAGTGGCTGCTGCCAGTGGCGATAAGTCGTGTCTTACCGGGTTGGACTCAAGAC
GATAGTTACCGGATAAGGCGCAGCGGTCGGGCTGAACGGGGGGTTCGTGCACACAGCCCAGCTTGGAGCGAACGACCTACACCGAACTGAGATACCTACA
GCGTGAGCTATGAGAAAGCGCCACGCTTCCCGAAGGGAGAAAGGCGGACAGGTATCCGGTAAGCGGCAGGGTCGGAACAGGAGAGCGCACGAGGGAGC
TTCCAGGGGGAAACGCCTGGTATCTTTATAGTCCTGTCGGGTTTCGCCACCTCTGACTTGAGCGTCGATTTTTGTGATGCTCGTCAGGGGGGCGGAGCCTAT
GGAAAAACGCCAGCAACGCGGCCTTTTTACGGTTCCTGGCCTTTTGCTGGCCTTTTGCTCACATGTTCTTTCCTGCGTTATCCCCTGATTCTGTGGATAACCGT
ATTACCGCCATGCATTAGTT

mEos2-Alpha-Actinin-19 plasmid sequence
Length: 7384 bp
Reference: Kanchanawong et al. [13]
TATTACCGCCATGCATTAGTTATTAATAGTAATCAATTACGGGGTCATTAGTTCATAGCCCATATATGGAGTTCCGCGTTACATAACTTACGGTAAATGGCCC
GCCTGGCTGACCGCCCAACGACCCCCGCCCATTGACGTCAATAATGACGTATGTTCCCATAGTAACGCCAATAGGGACTTTCCATTGACGTCAATGGGTGGA

GTATTTACGGTAAACTGCCCACTTGGCAGTACATCAAGTGTATCATATGCCAAGTACGCCCCCTATTGACGTCAATGACGGTAAATGGCCCGCCTGGCATTAT
GCCCAGTACATGACCTTATGGGACTTTCCTACTTGGCAGTACATCTACGTATTAGTCATCGCTATTACCATGGTGATGCGGTTTTGGCAGTACATCAATGGGC
GTGGATAGCGGTTTGACTCACGGGGATTTCCAAGTCTCCACCCCATTGACGTCAATGGGAGTTTGTTTTGGCACCAAAATCAACGGGACTTTCCAAAATGTC
GTAACAACTCCGCCCCATTGACGCAAATGGGCGGTAGGCGTGTACGGTGGGAGGTCTATATAAGCAGAGCTGGTTTAGTGAACCGTCAGATCCGCTAGCG
CTACCGGACTCAGATCTCGAGCTCAAGCTTCGCACCATCATGGACCATTATGATTCTCAGCAAACCAACGATTACATGCAGCCAGAAGAGGACTGGGACCG
GGACCTGCTCCTGGACCCGGCCTGGGAGAAGCAGCAGAGAAAGACATTCACGGCATGGTGTAACTCCCACCTCCGGAAGGCGGGGACACAGATCGAGAA
CATCGAAGAGGACTTCCGGGATGGCCTGAAGCTCATGCTGCTGCTGGAGGTCATCTCAGGTGAACGCTTGGCCAAGCCAGAGCGAGGCAAGATGAGAGTG
CACAAGATCTCCAACGTCAACAAGGCCCTGGATTTCATAGCCAGCAAAGGCGTCAAACTGGTGTCCATCGGAGCCGAAGAAATCGTGGATGGGAATGTGA
AGATGACCCTGGGCATGATCTGGACCATCATCCTGCGCTTTGCCATCCAGGACATCTCCGTGGAAGAGACTTCAGCCAAGGAAGGGCTGCTCCTGTGGTGT
CAGAGAAAGACAGCCCCTTACAAAAATGTCAACATCCAGAACTTCCACATAAGCTGGAAGGATGGCCTCGGCTTCTGTGCTTTGATCCACCGACACCGGCCC
GAGCTGATTGACTACGGGAAGCTGCGGAAGGATGATCCACTCACAAATCTGAATACGGCTTTTGACGTGGCAGAGAAGTACCTGGACATCCCCAAGATGCT
GGATGCCGAAGACATCGTTGGAACTGCCCGACCGGATGAGAAAGCCATCATGACTTACGTGTCTAGCTTCTACCACGCCTTCTCTGGAGCCCAGAAGGCGG
AGACAGCAGCCAATCGCATCTGCAAGGTGTTGGCCGTCAACCAGGAGAACGAGCAGCTTATGGAAGACTACGAGAAGCTGGCCAGTGATCTGTTGGAGTG
GATCCGCCGCACAATCCCGTGGCTGGAGAACCGGGTGCCCGAGAACACCATGCATGCCATGCAACAGAAGCTGGAGGACTTCCGGGACTACCGGCGCCTG
CACAAGCCGCCCAAGGTGCAGGAGAAGTGCCAGCTGGAGATCAACTTCAACACGCTGCAGACCAAGCTGCGGCTCAGCAACCGGCCTGCCTTCATGCCCTC
TGAGGGCAGGATGGTCTCGGACATCAACAATGCCTGGGGCTGCCTGGAGCAGGTGGAGAAGGGCTATGAGGAGTGGTTGCTGAATGAGATCCGGAGGCT
GGAGCGACTGGACCACCTGGCAGAGAAGTTCCGGCAGAAGGCCTCCATCCACGAGGCCTGGACTGACGGCAAAGAGGCCATGCTGCGACAGAAGGACTA
TGAGACCGCCACCCTCTCGGAGATCAAGGCCTTGCTCAAGAAGCATGAGGCCTTCGAGAGTGACCTGGCTGCCCACCAGGACCGTGTGGAGCAGATTGCC
GCCATCGCACAGGAGCTCAATGAGCTGGACTATTATGACTCACCCAGTGTCAACGCCCGTTGCCAAAAGATCTGTGACCAGTGGGACAATCTGGGGGCCCT
AACTCAGAAGCGAAGGGAAGCTCTGGAGCGGACCGAGAAACTGCTGGAGACCATTGACCAGCTGTACTTGGAGTATGCCAAGCGGGCTGCACCCTTCAAC
AACTGGATGGAGGGGGCCATGGAGGACCTGCAGGACACCTTCATTGTGCACACCATTGAGGAGATCCAGGGACTGACCACAGCCCATGAGCAGTTCAAGG
CCACCCTCCCTGATGCCGACAAGGAGCGCCTGGCCATCCTGGGCATCCACAATGAGGTGTCCAAGATTGTCCAGACCTACCACGTCAATATGGCGGGCACC
AACCCCTACACAACCATCACGCCTCAGGAGATCAATGGCAAATGGGACCACGTGCGGCAGCTGGTGCCTCGGAGGGACCAAGCTCTGACGGAGGAGCATG
CCCGACAGCAGCACAATGAGAGGCTACGCAAGCAGTTTGGAGCCCAGGCCAATGTCATCGGGCCCTGGATCCAGACCAAGATGGAGGAGATCGGGAGGA
TCTCCATTGAGATGCATGGGACCCTGGAGGACCAGCTCAGCCACCTGCGGCAGTATGAGAAGAGCATCGTCAACTACAAGCCAAAGATTGATCAGCTGGAG
GGCGACCACCAGCTCATCCAGGAGGCGCTCATCTTCGACAACAAGCACACCAACTACACCATGGAGCACATCCGTGTGGGCTGGGAGCAGCTGCTCACCAC
CATCGCCAGGACCATCAATGAGGTAGAGAACCAGATCCTGACCCGGGATGCCAAGGGCATCAGCCAGGAGCAGATGAATGAGTTCCGGGCCTCCTTCAAC
CACTTTGACCGGGATCACTCCGGCACACTGGGTCCCGAGGAGTTCAAAGCCTGCCTCATCAGCTTGGGTTATGATATTGGCAACGACCCCCAGGGAGAAGC
AGAATTTGCCCGCATCATGAGCATTGTGGACCCCAACCGCCTGGGGGTAGTGACATTCCAGGCCTTCATTGACTTCATGTCCCGCGAGACAGCCGACACAGA
TACAGCAGACCAAGTCATGGCTTCCTTCAAGATCCTGGCTGGGGACAAGAACTACATTACCATGGACGAGCTGCGCCGCGAGCTGCCACCCGACCAGGCTG
AGTACTGCATCGCGCGGATGGCCCCCTACACCGGCCCCGACTCCGTGCCAGGTGCTCTGGACTACATGTCCTTCTCCACGGCGCTGTACGGCGAGAGTGACC
TCAAGCTTCGAATTCTGCAGTCGACGGTACCGCGGGCCCGGGATCCACCGGTCGCCACCATGAGTGCGATTAAGCCAGACATGAAGATCAAACTCCGTATG
GAAGGCAACGTAAACGGGCACCACTTTGTGATCGACGGAGATGGTACAGGCAAGCCTTTTGAGGGAAAACAGAGTATGGATCTTGAAGTCAAAGAGGGC
GGACCTCTGCCTTTTGCCTTTGATATCCTGACCACTGCATTCCATTACGGCAACAGGGTATTCGCCAAATATCCAGACAACATACAAGACTATTTTAAGCAGT
CGTTTCCTAAGGGGTATTCGTGGGAACGAAGCTTGACTTTCGAAGACGGGGGCATTTGCATTGCCAGAAACGACATAACAATGGAAGGGGACACTTTCTAT
AATAAAGTTCGATTTTATGGTACCAACTTTCCCGCCAATGGTCCAGTTATGCAGAAGAAGACGCTGAAATGGGAGCCCTCCACTGAGAAAATGTATGTGCGT
GATGGAGTGCTGACGGGTGATATTCATATGGCTTTGTTGCTTGAAGGAAATGCCCATTACCGATGTGACTTCAGAACTACTTACAAAGCTAAGGAGAAGGG
TGTCAAGTTACCAGGCTACCACTTTGTGGACCACTGCATTGAGATTTTAAGCCATGACAAAGATTACAACAAGGTTAAGCTGTATGAGCATGCTGTTGCTCAT
TCTGGATTGCCTGACAATGCCAGACGATAAGCGGCCGCGACTCTAGATCATAATCAGCCATACCACATTTGTAGAGGTTTTACTTGCTTTAAAAAACCTCCCA
CACCTCCCCCTGAACCTGAAACATAAAATGAATGCAATTGTTGTTGTTAACTTGTTTATTGCAGCTTATAATGGTTACAAATAAAGCAATAGCATCACAAATTT
CACAAATAAAGCATTTTTTTCACTGCATTCTAGTTGTGGTTTGTCCAAACTCATCAATGTATCTTAAGGCGTAAATTGTAAGCGTTAATATTTTGTTAAAATTC
GCGTTAAATTTTTGTTAAATCAGCTCATTTTTTAACCAATAGGCCGAAATCGGCAAAATCCCTTATAAATCAAAAGAATAGACCGAGATAGGGTTGAGTGTTG
TTCCAGTTTGGAACAAGAGTCCACTATTAAAGAACGTGGACTCCAACGTCAAAGGGCGAAAAACCGTCTATCAGGGCGATGGCCCACTACGTGAACCATCA
CCCTAATCAAGTTTTTTGGGGTCGAGGTGCCGTAAAGCACTAAATCGGAACCCTAAAGGGAGCCCCCGATTTAGAGCTTGACGGGGAAAGCCGGCGAACG
TGGCGAGAAAGGAAGGGAAGAAAGCGAAAGGAGCGGGCGCTAGGGCGCTGGCAAGTGTAGCGGTCACGCTGCGCGTAACCACCACACCCGCCGCGCTT
AATGCGCCGCTACAGGGCGCGTCAGGTGGCACTTTTCGGGGAAATGTGCGCGGAACCCCTATTTGTTTATTTTTCTAAATACATTCAAATATGTATCCGCTCA
TGAGACAATAACCCTGATAAATGCTTCAATAATATTGAAAAAGGAAGAGTCCTGAGGCGGAAAGAACCAGCTGTGGAATGTGTGTCAGTTAGGGTGTGGA
AAGTCCCCAGGCTCCCCAGCAGGCAGAAGTATGCAAAGCATGCATCTCAATTAGTCAGCAACCAGGTGTGGAAAGTCCCCAGGCTCCCCAGCAGGCAGAA
GTATGCAAAGCATGCATCTCAATTAGTCAGCAACCATAGTCCCGCCCCTAACTCCGCCCATCCCGCCCCTAACTCCGCCCAGTTCCGCCCATTCTCCGCCCCAT
GGCTGACTAATTTTTTTTATTTATGCAGAGGCCGAGGCCGCCTCGGCCTCTGAGCTATTCCAGAAGTAGTGAGGAGGCTTTTTTGGAGGCCTAGGCTTTTGC
AAAGATCGATCAAGAGACAGGATGAGGATCGTTTCGCATGATTGAACAAGATGGATTGCACGCAGGTTCTCCGGCCGCTTGGGTGGAGAGGCTATTCGGC
TATGACTGGGCACAACAGACAATCGGCTGCTCTGATGCCGCCGTGTTCCGGCTGTCAGCGCAGGGGCGCCCGGTTCTTTTTGTCAAGACCGACCTGTCCGGT
GCCCTGAATGAACTGCAAGACGAGGCAGCGCGGCTATCGTGGCTGGCCACGACGGGCGTTCCTTGCGCAGCTGTGCTCGACGTTGTCACTGAAGCGGGAA
GGGACTGGCTGCTATTGGGCGAAGTGCCGGGGCAGGATCTCCTGTCATCTCACCTTGCTCCTGCCGAGAAAGTATCCATCATGGCTGATGCAATGCGGCGG
CTGCATACGCTTGATCCGGCTACCTGCCCATTCGACCACCAAGCGAAACATCGCATCGAGCGAGCACGTACTCGGATGGAAGCCGGTCTTGTCGATCAGGA
TGATCTGGACGAAGAGCATCAGGGGCTCGCGCCAGCCGAACTGTTCGCCAGGCTCAAGGCGAGCATGCCCGACGGCGAGGATCTCGTCGTGACCCATGGC
GATGCCTGCTTGCCGAATATCATGGTGGAAAATGGCCGCTTTTCTGGATTCATCGACTGTGGCCGGCTGGGTGTGGCGGACCGCTATCAGGACATAGCGTT
GGCTACCCGTGATATTGCTGAAGAGCTTGGCGGCGAATGGGCTGACCGCTTCCTCGTGCTTTACGGTATCGCCGCTCCCGATTCGCAGCGCATCGCCTTCTA
TCGCCTTCTTGACGAGTTCTTCTGAGCGGGACTCTGGGGTTCGAAATGACCGACCAAGCGACGCCCAACCTGCCATCACGAGATTTCGATTCCACCGCCGCC
TTCTATGAAAGGTTGGGCTTCGGAATCGTTTTCCGGGACGCCGGCTGGATGATCCTCCAGCGCGGGGATCTCATGCTGGAGTTCTTCGCCCACCCTAGGGG
GAGGCTAACTGAAACACGGAAGGAGACAATACCGGAAGGAACCCGCGCTATGACGGCAATAAAAAGACAGAATAAAACGCACGGTGTTGGGTCGTTTGTT
CATAAACGCGGGGTTCGGTCCCAGGGCTGGCACTCTGTCGATACCCCACCGAGACCCCATTGGGGCCAATACGCCCGCGTTTCTTCCTTTTCCCCACCCCACC
CCCCAAGTTCGGGTGAAGGCCCAGGGCTCGCAGCCAACGTCGGGGCGGCAGGCCCTGCCATAGCCTCAGGTTACTCATATATACTTTAGATTGATTTAAAA
CTTCATTTTTAATTTAAAAGGATCTAGGTGAAGATCCTTTTTGATAATCTCATGACCAAAATCCCTTAACGTGAGTTTTCGTTCCACTGAGCGTCAGACCCCGT
AGAAAAGATCAAAGGATCTTCTTGAGATCCTTTTTTTCTGCGCGTAATCTGCTGCTTGCAAACAAAAAAACCACCGCTACCAGCGGTGGTTTGTTTGCCGGAT
CAAGAGCTACCAACTCTTTTTCCGAAGGTAACTGGCTTCAGCAGAGCGCAGATACCAAATACTGTTCTTCTAGTGTAGCCGTAGTTAGGCCACCACTTCAAG

AACTCTGTAGCACCGCCTACATACCTCGCTCTGCTAATCCTGTTACCAGTGGCTGCTGCCAGTGGCGATAAGTCGTGTCTTACCGGGTTGGACTCAAGACGAT
AGTTACCGGATAAGGCGCAGCGGTCGGGCTGAACGGGGGGTTCGTGCACACAGCCCAGCTTGGAGCGAACGACCTACACCGAACTGAGATACCTACAGCG
TGAGCTATGAGAAAGCGCCACGCTTCCCGAAGGGAGAAAGGCGGACAGGTATCCGGTAAGCGGCAGGGTCGGAACAGGAGAGCGCACGAGGGAGCTTC
CAGGGGGAAACGCCTGGTATCTTTATAGTCCTGTCGGGTTTCGCCACCTCTGACTTGAGCGTCGATTTTTGTGATGCTCGTCAGGGGGGCGGAGCCTATGG
AAAAACGCCAGCAACGCGGCCTTTTTACGGTTCCTGGCCTTTTGCTGGCCTTTTGCTCACATGTTCTTTCCTGCGTTATCCCCTGATTCTGTGGATAACCG

Supplementary Table 1. Plasmid sequences used in this study.

References

1. Descloux, A., Grußmayer, K. S. & Radenovic, A. Parameter-free image resolution estimation based on decorrelation analysis. *Nat. Methods* 16, 918–924 (2019).

2. Ovesný, M., Křížek, P., Borkovec, J., Svindrych, Z. & Hagen, G. M. ThunderSTORM: a comprehensive ImageJ plug-in for PALM and STORM data analysis and super-resolution imaging. *Bioinformatics* 30, 2389–90 (2014).

3. Marsh, R. J. *et al.* Artifact-free high-density localization microscopy analysis. *Nat. Methods* 15, 689–692 (2018).

4. Leitao, S. M. *et al.* Time-resolved scanning ion conductance microscopy for three-dimensional tracking of nanoscale cell surface dynamics 2. *bioRxiv* 2021.05.13.444009 (2021) doi:10.1101/2021.05.13.444009.

5. Steinbock, L. J., Bulushev, R. D., Krishnan, S., Raillon, C. & Radenovic, A. DNA translocation through low-noise glass nanopores. *ACS Nano* 7, 11255–11262 (2013).

6. Navikas, V. *et al.* High-Throughput, Nanocapillary Filling Method Based on Microwave Radiation. *ACS Appl. Nano Mater.* acsanm.0c01345 (2020) doi:10.1021/acsanm.0c01345.

7. Werther, P. *et al.* Live-Cell Localization Microscopy with a Fluorogenic and Self-Blinking Tetrazine Probe. *Angew. Chemie Int. Ed.* 59, 804–810 (2020).

8. Werther, P. *et al.* Bioorthogonal red and far-red fluorogenic probes for wash-free live-cell and super-resolution microscopy. *bioRxiv* 2020.08.07.241687 (2020) doi:10.1101/2020.08.07.241687.

9. Textor, M. & Grandin, H. M. *Intelligent surfaces in biotechnology : scientific and engineering concepts, enabling technologies, and translation to bio-oriented applications*. (John Wiley & Sons,

2012).

10. Meimetis, L. G., Carlson, J. C. T., Giedt, R. J., Kohler, R. H. & Weissleder, R. Ultrafluorogenic coumarin-tetrazine probes for real-time biological imaging. *Angew. Chemie - Int. Ed.* 53, 7531–7534 (2014).

11. Geissbuehler, S. *et al.* Mapping molecular statistics with balanced super-resolution optical fluctuation imaging (bSOFI). *Opt. Nanoscopy* 1, 1–7 (2012).

12. Izeddin, I. *et al.* Super-Resolution Dynamic Imaging of Dendritic Spines Using a Low-Affinity Photoconvertible Actin Probe. *PLoS One* 6, e15611 (2011).

13. Kanchanawong, P. *et al.* Nanoscale architecture of integrin-based cell adhesions. *Nature* 468, 580–584 (2010).
