# Supplementary material for: Correlative 3D microscopy of single cells using super-resolution and scanning ion-conductance microscopy
Source: Nat Commun. 2021 Jul 27;12:4565. doi: 10.1038/s41467-021-24901-3 (PMC8316521; doi:10.1038/s41467-021-24901-3)
Supplement: Supplementary file 2 — Reporting Summary [file 41467_2021_24901_MOESM2_ESM.pdf]

nature research

Corresponding author(s): Georg E. Fantner and Aleksandra Radenovic

Last updated by author(s): 30.06.2021.

Reporting Summary

Nature Research wishes to improve the reproducibility of the work that we publish. This form provides structure for consistency and transparency in reporting. For further information on Nature Research policies, see our Editorial Policies and the Editorial Policy Checklist.

Statistics

For all statistical analyses, confirm that the following items are present in the figure legend, table legend, main text, or Methods section.

n/a	Confirmed	
☐	☒	The exact sample size (*n*) for each experimental group/condition, given as a discrete number and unit of measurement
☐	☒	A statement on whether measurements were taken from distinct samples or whether the same sample was measured repeatedly
☐	☒	The statistical test(s) used AND whether they are one- or two-sided<br>*Only common tests should be described solely by name; describe more complex techniques in the Methods section.*
☒	☐	A description of all covariates tested
☒	☐	A description of any assumptions or corrections, such as tests of normality and adjustment for multiple comparisons
☐	☒	A full description of the statistical parameters including central tendency (e.g. means) or other basic estimates (e.g. regression coefficient) AND variation (e.g. standard deviation) or associated estimates of uncertainty (e.g. confidence intervals)
☐	☒	For null hypothesis testing, the test statistic (e.g. *F*, *t*, *r*) with confidence intervals, effect sizes, degrees of freedom and *P* value noted<br>*Give P values as exact values whenever suitable.*
☒	☐	For Bayesian analysis, information on the choice of priors and Markov chain Monte Carlo settings
☒	☐	For hierarchical and complex designs, identification of the appropriate level for tests and full reporting of outcomes
☒	☐	Estimates of effect sizes (e.g. Cohen's *d*, Pearson's *r*), indicating how they were calculated

Our web collection on statistics for biologists contains articles on many of the points above.

Software and code

Policy information about availability of computer code

Data collection	All presented image data were acquired on a custom-built microscopes as described in the paper. Custom codes written in LabVIEW (version 18.0) were used to operate each setup. Micro manager (version 1.4.22) was used to record images on SOFI 2D setup and on combined SICM-SOFI system. RAW and processed data used to generate the main figures of the manuscript are available:
Data analysis	SOFI data analysis was performed with an algorithm implemented in MATLAB 2017b(version 9.3.0.713579). SICM data was processed with Gwydion (version 2.56). Images then were registered with a script written in Python (3.7.3). All image data was further processed with Fiji (ImageJ, version 1.52s). 3D rendering was performed with Blender 3D software (version 2.83). 2D/3D SOFI code used for image processing are deposited in public repository: https://github.com/kgrussmayer/sofipackage.

For manuscripts utilizing custom algorithms or software that are central to the research but not yet described in published literature, software must be made available to editors and reviewers. We strongly encourage code deposition in a community repository (e.g. GitHub). See the Nature Research guidelines for submitting code & software for further information.

Data

Policy information about availability of data

All manuscripts must include a data availability statement. This statement should provide the following information, where applicable:

- Accession codes, unique identifiers, or web links for publicly available datasets
- A list of figures that have associated raw data
- A description of any restrictions on data availability

All raw and processed data, used to generate figures presented in the paper are available:

Field-specific reporting

Please select the one below that is the best fit for your research. If you are not sure, read the appropriate sections before making your selection.

- [x] Life sciences
- [ ] Behavioural & social sciences
- [ ] Ecological, evolutionary & environmental sciences

For a reference copy of the document with all sections, see nature.com/documents/nr-reporting-summary-flat.pdf

Life sciences study design

All studies must disclose on these points even when the disclosure is negative.

Sample size	Sample sizes was not predetermined based on statistical calculations, because no hypothesis-based experiment was performed. 2 separate samples were used with a six cells randomly selected cells imaged in a correlative manner from each of the fixed samples with stained COS-7 cells with a standard staining protocol. 16-30k frames were recorded for each color channel for 2D SOFI computation and 8k frames were recorded for 3D SOFI computation. 300 SOFI frames were recorded for live-cell measurements unless stated differently. 5 samples of transected COS-7 cells were used in a paper. Live cells were selected for imaging based on a presence of expression of protein of interest.
Data exclusions	Fixed samples that stained in a manner inconsistent with published literature due to handling errors were excluded.
Replication	All performed replications were successful. We used a known and previously published staining protocols and performed imaging on 2 separate samples processed a few weeks apart.
Randomization	No randomization was used for experimental data. There is no group allocation component in our study.
Blinding	There is no group allocation component in our study.

Reporting for specific materials, systems and methods

We require information from authors about some types of materials, experimental systems and methods used in many studies. Here, indicate whether each material, system or method listed is relevant to your study. If you are not sure if a list item applies to your research, read the appropriate section before selecting a response.

Materials & experimental systems

n/a	Involved in the study	
☐	☒	Antibodies
☐	☒	Eukaryotic cell lines
☒	☐	Palaeontology and archaeology
☒	☐	Animals and other organisms
☒	☐	Human research participants
☒	☐	Clinical data
☒	☐	Dual use research of concern

Methods

n/a	Involved in the study	
☒	☐	ChIP-seq
☒	☐	Flow cytometry
☒	☐	MRI-based neuroimaging

Antibodies

Antibodies used	Anti alpha-tubulin antibody (clone B-5-1-2 ascites fluid, T5168 , Lot 047M4760V, Sigma-Aldrich) and donkey anti-Mouse IgG (H+L) Highly Cross-Adsorbed Secondary Antibody (labeled with Abberior FLIP-565-NHS (Abberior) and purified as described in Methods section, A16019, Lot 28-167-092613, Invitrogen).
Validation	Anti alpha-tubulin antibody (clone B-5-1-2 mouse monoclonal ascites fluid, T5168 , Sigma-Aldrich) used in COS-7 (African green monkey) cells. The manufacture's website shows validation that the antibody is specific for African green monkey protein. The antibody is highly cited for immunofluorescence according to citeab.com.

Eukaryotic cell lines

Policy information about cell lines

Cell line source(s)	COS-7 cells (ECACC 87021302) obtained from ECACC, were a kind gift of the Manley lab (EPFL).
Authentication	COS-7 cell line was not authenticated.
Mycoplasma contamination	COS-7 cell line was not tested for Mycoplasma contamination.
Commonly misidentified lines (See ICLAC register)	We did not use commonly misidentified cell lines, the only cell line used in this study was COS-7.
