# Supplementary material for: Correlative 3D microscopy of single cells using super-resolution and scanning ion-conductance microscopy
Source: Nat Commun. 2021 Jul 27;12:4565. doi: 10.1038/s41467-021-24901-3 (PMC8316521; doi:10.1038/s41467-021-24901-3)
Supplement: Supplementary file 5 — Description of Additional Supplementary Files [file 41467_2021_24901_MOESM5_ESM.pdf]

Title: Supplementary Movie 1.
Description: 3D rendering of correlative SICM and 3D SOFI data. The tubulin and actin 3D SOFI channels are rendered as volumes consisting of 22 planes. SICM information is rendered as topography correlated with fluorescence data.

Title: Supplementary Movie 2.
Description 3D rendering of correlative SICM and 2D SOFI, visualizing the dynamics of actinin in live COS-7 cells.
